# Biochemical reconstitution of sister chromatid cohesion establishment during DNA replication

**DOI:** 10.1016/j.molcel.2025.08.026

**Published:** 2025-09-16

**Authors:** Masashi Minamino, John F.X. Diffley, Frank Uhlmann

**Affiliations:** 1 Chromosome Segregation Laboratory, The Francis Crick Institute, London NW1 1AT, UK; 2 Research Division, Chugai Pharmaceutical Co. Ltd., Yokohama 244-8602, Japan; 3 Chromosome Replication Laboratory, The Francis Crick Institute, London NW1 1AT, UK

## Abstract

Concomitant with DNA replication, the ring-shaped cohesin complex encircles both newly synthesized sister chromatids, enabling their faithful segregation during cell divisions. Our molecular understanding of how cohesin co-entraps both replication products remains incomplete. Here, we reconstitute sister chromatid cohesion establishment using purified budding yeast proteins. Cohesin rings, initially loaded onto template DNA, remain DNA bound during complete DNA synthesis. Some of these cohesin rings encircle both sister chromatids, consistent with the idea that replisomes traverse through cohesin rings. Often, however, cohesin ends up embracing only one of the two replication products, suggesting that a two-step capture mechanism operates during cohesion establishment. Additionally, DNA replication initiates new cohesin recruitment as a further means to generate sister chromatid cohesion. Our results illustrate that more than one pathway leads to sister chromatid cohesion, and they make cohesion establishment amenable to direct biochemical exploration.

## Graphical abstract

**Graphical abstract:**
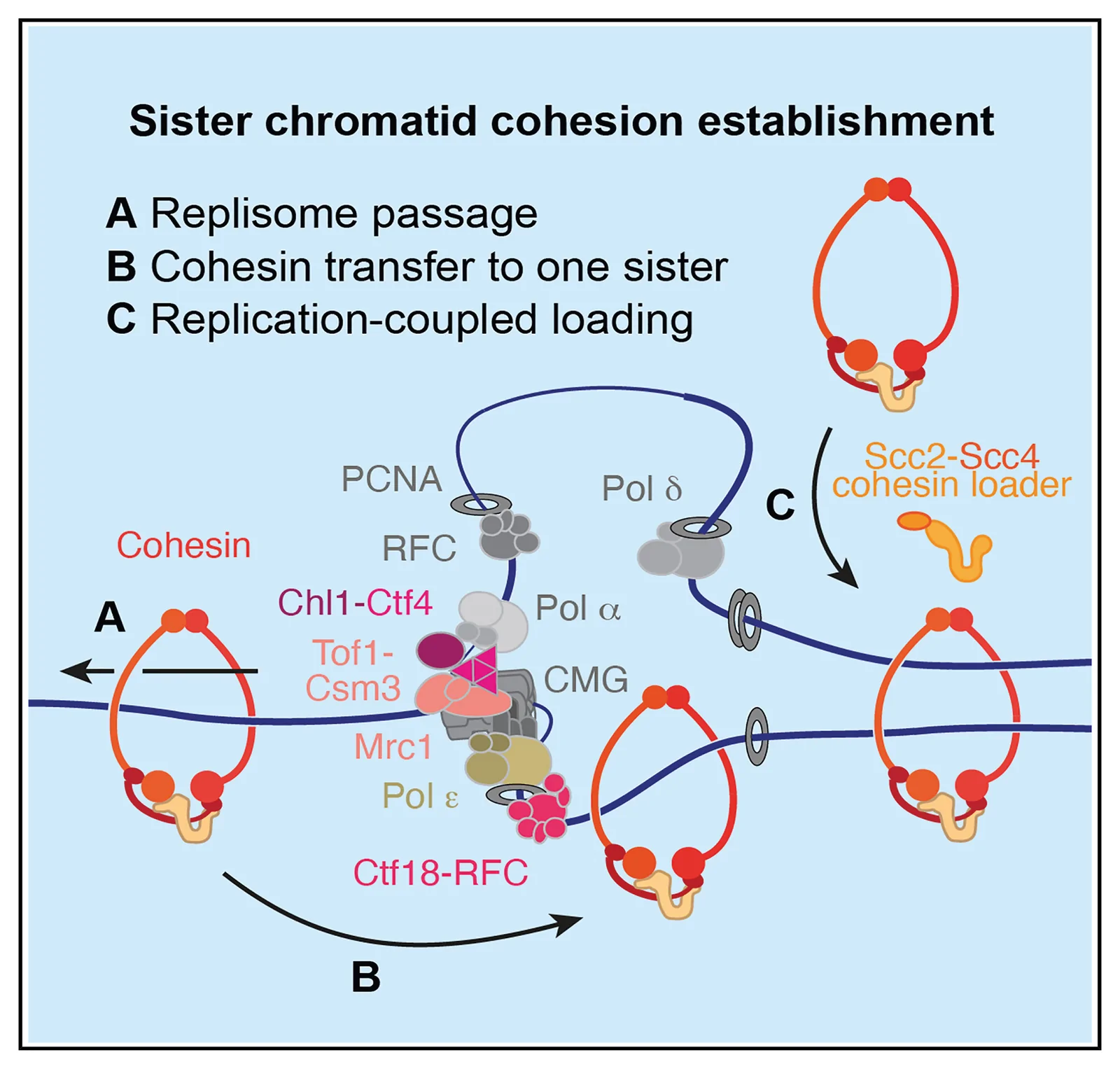

## Introduction

Concurrently with genome replication during the S phase of the eukaryotic cell cycle, the chromosomal cohesin complex establishes physical links between the two replication products.^1–4^ These links are known as sister chromatid cohesion. They provide the counterforce to the mitotic spindle when chromosomes align on the cell equator, before cohesin cleavage triggers chromosome segregation into daughter cells in anaphase. Cohesin is a ring-shaped protein complex of the structural maintenance of chromosomes (SMC) family.^5^ Cohesin rings are topologically loaded onto DNA already before the onset of DNA replication, during the G1 phase, with the help of an Scc2–Scc4 cohesin loader complex.^6–8^ Following DNA replication, single cohesin rings embrace both sister chromatids.^9–11^ What happens during DNA replication, when the replisome moves along chromosomes and encounters cohesin, remains incompletely understood. Different scenarios have been proposed for how cohesin transitions from encircling one DNA to embracing two sister DNAs. These possibilities are not mutually exclusive, and they include (1) replisome passage through cohesin rings,^12,13^ (2) transient unloading and then two-step capture of both sister DNAs behind the fork,^10,13^ (3) *de novo* cohesin recruitment to the replication fork,^7,10^ and (4) cohesin pushing by the replisome and cohesion establishment at sites of replication termination.^14^

In addition to the cohesin complex, a series of replisome components contribute to sister chromatid cohesion establishment, known as “cohesion establishment factors.” They include the Tof1-Csm3-Mrc1 replisome progression complex,^15–17^ the structural replisome component Ctf4 with its binding partner, the Chl1 helicase,^18–20^ as well as the PCNA sliding clamp and its Ctf18-RFC clamp loader.^18,21,22^ Complex genetic relationships between these cohesion establishment factors^7,23,24^ suggest that more than one reaction contributes to efficient sister chromatid cohesion establishment.

In addition to entrapping both replication products, cohesin becomes acetylated on two conserved lysine residues to ensure that sister chromatid co-entrapment results in enduring sister chromatid cohesion.^25,26^ Acetylation stabilizes sister chromatid linkages by stopping the dynamic cohesin loading and unloading cycles that otherwise characterize cohesin’s chromatin interactions.^27–30^ The acetylation reaction is temporally and spatially controlled by its dependence on transient DNA structures that form during Okazaki fragment maturation,^31^ thereby directing acetylation to newly established sister chromatid cohesion. All of the above-mentioned cohesion establishment factors, directly or indirectly, facilitate the cohesin acetylation reaction in as yet unknown ways.^24,32^

Biochemical reconstitution is a powerful approach to dissect and understand complex molecular events. Complete eukaryotic DNA replication has been reconstituted using purified components from budding yeast,^33,34^ as has the topological loading of cohesin rings onto DNA.^6,35^ Combining *in vitro* cohesin loading with DNA replication opens an opportunity to study the establishment of sister chromatid cohesion. A first report on such reconstituted replisome-cohesin encounters revealed that cohesin retains DNA association during replication and that Ctf4 and Chl1 promote cohesin loading at the fork.^36^ Whether cohesin topologically entrapped both replication products following *in vitro* DNA replication remained untested. Replisome-cohesin encounters were also studied in *Xenopus* cell-free egg extracts, where reported outcomes included replisome passage or cohesin-pushing events.^3,14,37^ The complex nature of the extracts precluded the molecular dissection of cohesion establishment and characterization of the resultant sister chromatid linkages.

Here, we reconstitute sister chromatid cohesion establishment during DNA replication using purified budding yeast proteins. Cohesin that is loaded onto DNA before the onset of DNA replication remains bound to DNA and topologically embraces both replication products. Additionally, the replisome promotes replication-coupled cohesin recruitment as a second means to establish sister chromatid cohesion. In both cases, however, we find that cohesin often entraps only one of the two DNA products, a pattern suggestive of a two-step sister chromatid capture mechanism. *In vitro* co-entrapment of both replication products occurs independently of replisome-associated cohesion establishment factors, suggesting that these factors play roles during *in vivo* chromatin replication or by promoting the cohesin acetylation reaction.

## Results

### Cohesin remains DNA bound during DNA replication

We set out to reconstitute sister chromatid cohesion establishment during DNA replication. We began by purifying all budding yeast components required for origin-dependent DNA replication.^33,34^ In our experiments, MCM helicase double hexamers were assembled onto an *ARS1* replication origin-containing, 3.2 kb, circular plasmid DNA template. Next, MCM double hexamers were activated by Dbf4-dependent kinase (DDK). Finally, origin firing factors, DNA polymerases, and accessory proteins were added to initiate DNA replication (Figure S1A). [α-^32^P]dCTP was included amongst the nucleotides to facilitate visualization of replication products. Following deproteination, the reactions were then resolved by native agarose gel electrophoresis, and replication products were visualized by autoradiography. Total DNA, including the template, was stained with the DNA dye SYBR Gold. Topoisomerase II (Top II) was included in our reactions to allow decatenation of fully replicated sister DNA circles. This setup resulted in the generation of completely replicated circular DNA products (Figure S1B).

**Figure 1:**
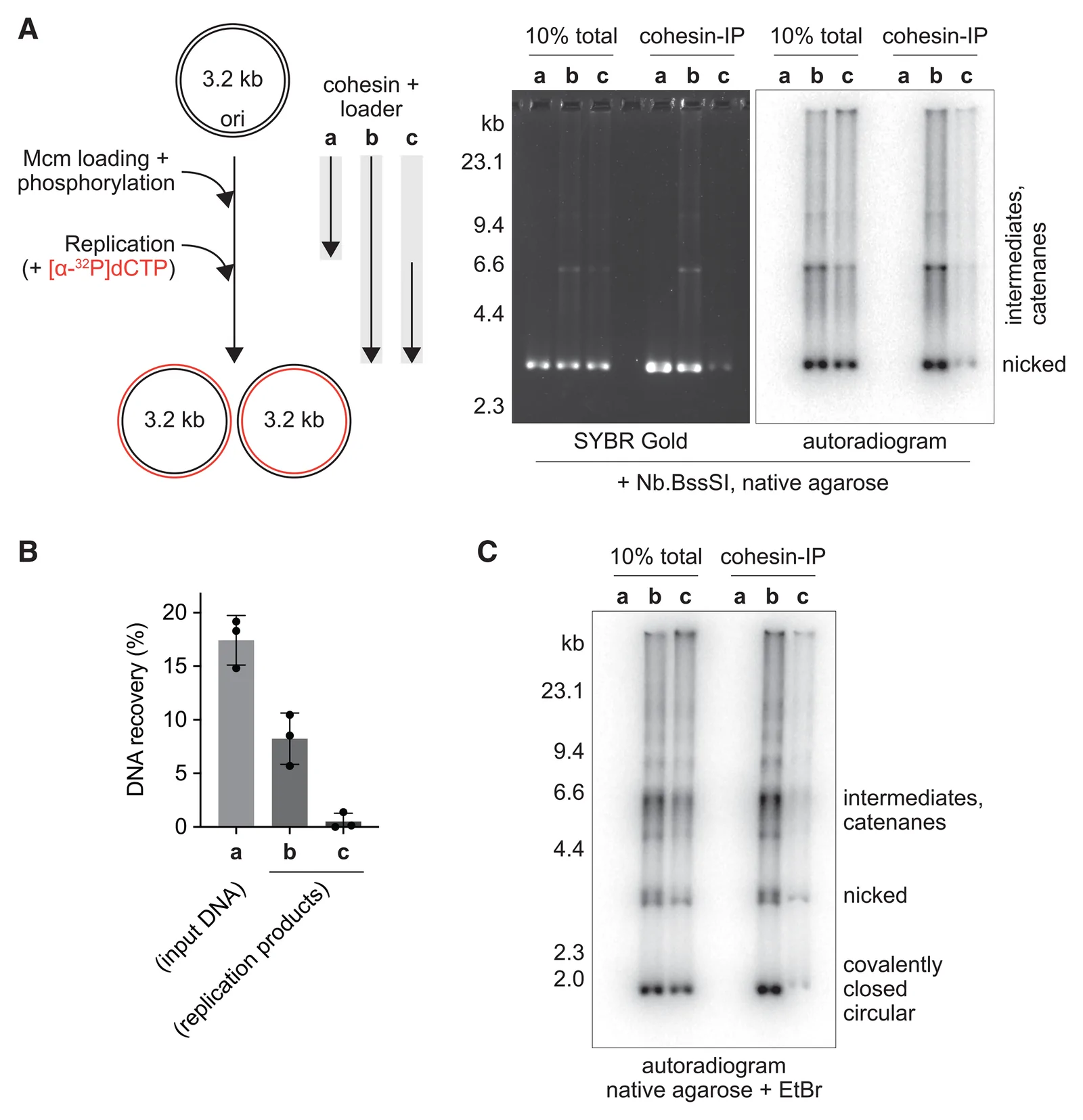
Cohesin remains DNA bound during DNA replication (A) Schematic of the experiment in which cohesin is “a” loaded onto unreplicated DNA during origin licensing, “b” loaded onto unreplicated DNA during origin licensing followed by DNA replication, and “c” added after origin licensing only during the replication reaction. Total products (total) and cohesin-bound DNAs (cohesin immunoprecipitation [IP]) were separated following nicking enzyme Nb.BssSI treatment by agarose gel electrophoresis. Total DNA was visualized by SYBR Gold staining, and replication products by autoradiography of incorporated [α-^2^P]dCTP. (B) The experiment in (A) was independently repeated three times, and the DNA recovery by cohesin of input DNA and replication products was quantified. Individual results are shown (black circles), and the means (bars) and standard deviations (error bars) are indicated. See also Figure S1 for further characterization of the DNA replication reaction. (C) The DNA samples from one of the experiments in (A) were separated, without nicking, on an ethidium bromide (EtBr)-containing agarose gel. Covalently closed circular DNAs migrate faster due to EtBr-induced supercoiling, confirming that cohesin remains bound to fully replicated sister DNAs. See also Figure S2 for characterization of the fully replicated species, as well as an experiment to probe the contribution of cohesion establishment factors to cohesin retention during DNA replication.

We next added cohesin to this replication assay. *In vivo*, cohesin is topologically loaded onto chromosomes before the onset of DNA replication.^4,7^ We therefore started by loading cohesin onto our template plasmid DNA, with the help of the cohesin loader and ATP, before MCM loading and activation. Cohesin immunoprecipitation at this stage, before initiating DNA synthesis, confirmed that cohesin was successfully loaded onto the template DNA (Figure 1A, sample **a**). We next initiated DNA replication using this cohesin-bound template by adding the remaining replication proteins, which included the cohesion establishment factors Ctf4, Tof1-Csm3, and Mrc1. We additionally included purified Chl1, Ctf18-RFC, and Pds5 and Eco1.^31^ Following DNA replication, cohesin immunoprecipitation revealed that cohesin was associated with circular replication products (Figure 1A, sample **b**). This observation left unresolved whether cohesin remained DNA bound during DNA replication or whether new cohesin was loaded during or following DNA synthesis. To distinguish between these two possibilities, we prepared sample **c**, in which no cohesin was loaded onto the DNA template, but cohesin and the cohesin loader were added during the DNA replication incubation. In this case, cohesin is barely associated with replication products (Figure 1A, sample **c**). The likely reason for no cohesin loading during DNA replication was the presence of 250 mM potassium glutamate in the replication buffer, high ionic strength conditions that disfavor cohesin loading. Therefore, cohesin bound to replication products in sample **b** must have derived from cohesin that was loaded onto DNA at the beginning of the experiment. These observations suggest that cohesin that encircles DNA remains DNA bound during DNA synthesis, a conclusion that was recently also reached by Murayama et al.^36^

We quantified the fraction of cohesin-bound DNA before DNA replication (stained with SYBR Gold) in three repeats of the experiment, as well as the fraction of cohesin-bound replication products (visualized by autoradiography). These measurements revealed that the proportion of replication products bound by cohesin was around half of the fraction of cohesin-bound template DNA (Figure 1B). Therefore, not all cohesin-bound DNAs retained cohesin association during *in vitro* DNA replication, an observation that we will return to below.

### Cohesin associates with completely replicated DNA circles

The above experiment showed that cohesin remains bound to a proportion of *in vitro* replicated DNA circles. While our template DNA is supercoiled, topoisomerases present in the replication reaction cause its partial relaxation. To facilitate the quantitative comparison between input and replicated DNAs, we had treated all samples in Figure 1A with a nicking enzyme, converting all DNAs into relaxed circles, before agarose gel electrophoresis. To investigate whether cohesin remains associated with completely replicated, covalently closed replication products, we omitted nicking enzyme treatment and instead resolved the replication products on an ethidium bromide-containing agarose gel. Ethidium bromide intercalates into DNA and thereby leads to supercoiling only of covalently closed circular DNAs, i.e., completely sealed circular DNA products without any remaining nicks. Our replication reaction proceeded to yield such covalently closed circular DNA replication products (Figure S2A), and these were bound by cohesin (Figure 1C). This observation demonstrates that cohesin that is loaded onto DNA before DNA replication can remain bound to DNA throughout all DNA synthesis, ligation, and decatenation steps that lead to two completely replicated sister DNA products.

We investigated whether cohesin retention during DNA synthesis requires any of the replisome-associated cohesion establishment factors. However, omitting Ctf4-Chl1, Tof1-Csm3, or Mrc1 from the replication reaction did not noticeably alter the efficiency with which cohesin was retained (Figure S2B), an observation corroborated by Murayama et al.^36^ Taken together, our results so far suggest that cohesin retains DNA binding during the process of complete *in vitro* plasmid DNA replication.

### Biochemical reconstitution of sister chromatid cohesion establishment

An important unanswered question from the above experiments is whether, following DNA replication, cohesin entrapped one or both replication products. Size analysis of the replication products, required to answer this question, is made difficult by the many DNA-binding proteins that are contained in the replication reaction and that prevent DNA entry during conventional gel electrophoresis. To circumvent this problem, we introduced a cohesin variant into our experiments that can be covalently circularized using cysteine-specific crosslinking (6C cohesin).^7^ 3 engineered cysteine pairs allow covalent closure of the three cohesin ring interfaces using the bi-cysteine crosslinker bis-maleimidoethane (BMOE), while a control 5C variant lacks one of these cysteines. We confirmed that 6C cohesin could be efficiently crosslinked and that crosslinking following topological loading resulted in SDS-resistant retention of 6C cohesin, but not 5C cohesin, on DNA (Figures S3A–S3C). The SDS-resistant nature of 6C cohesin’s DNA entrapment allowed us to use this detergent to wash off all other proteins from the replication products and then analyze the cohesion status by gel electrophoresis.

**Figure 2:**
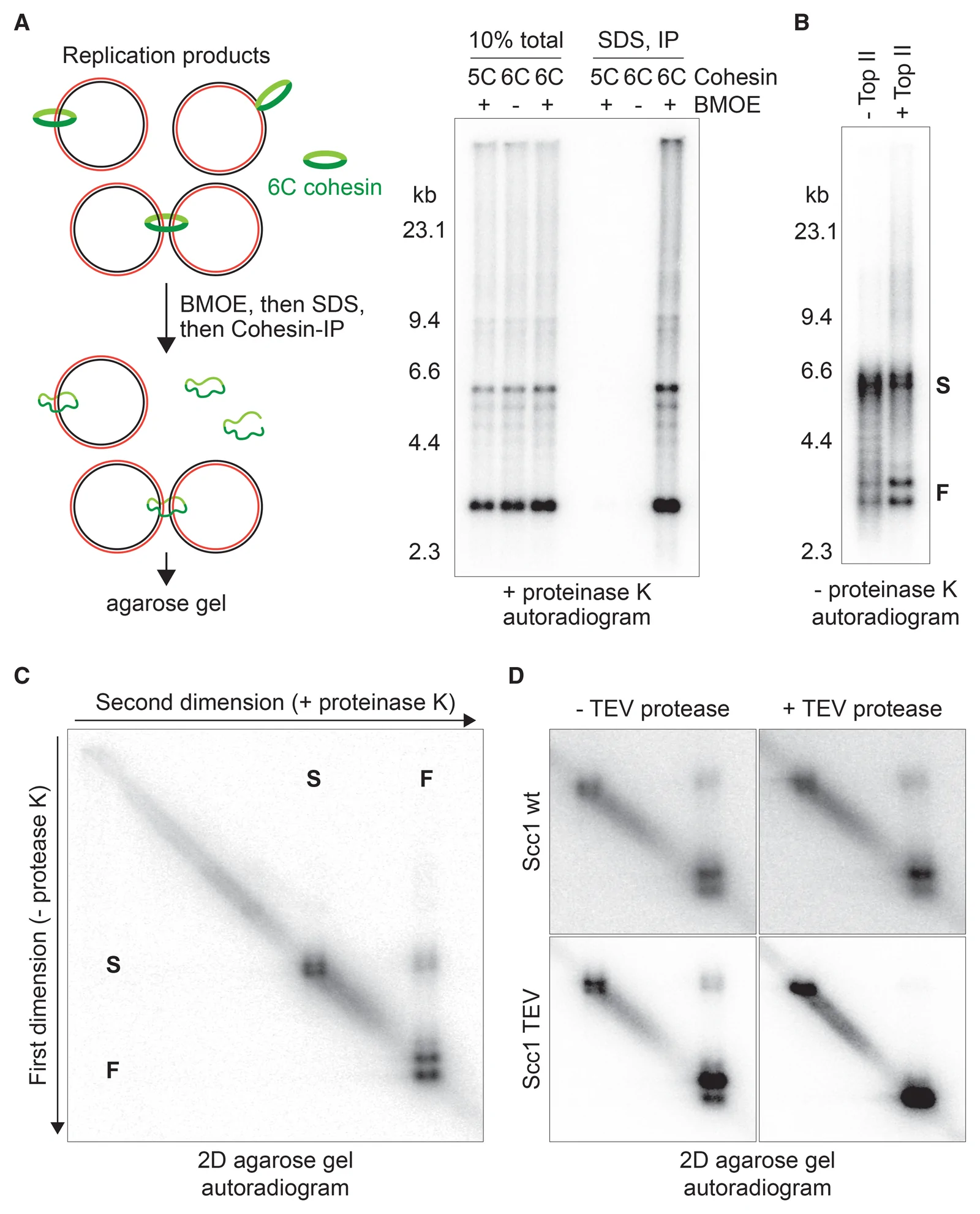
Biochemical reconstitution of sister chromatid cohesion establishment (A) Schematic of replication products bound by 6C cohesin and the consequences of its crosslinking and denaturation. Autoradiogram of total replication products formed from 5C or 6C cohesin-bound template DNA (total) and of cohesin IP following SDS denaturation. (B) Replication product sizes following 6C cohesin crosslinking. DNA replication was performed in the presence or absence of Top II. Fast-migrating products “F” of approximately the size of the 3.2 kb plasmid, as well as slow-migrating products “S” of around double the size, are indicated (the Pif1 helicase was added to this reaction to facilitate replication termination without Top II^38^). (C) Two-dimensional (2D) gel electrophoresis following replication of a 6C cohesin-bound template DNA, crosslinking, denaturation, and cohesin IP. The first dimension was native gel electrophoresis, and the second dimension included migration through Proteinase K and SDS. (D) As in (C), but 6C cohesin without or with an Scc1 TEV protease recognition site was used, and samples were treated without or with TEV protease before 2D gel electrophoresis. Scc1 TEV cleavage resolved protein-dependent dimers, as well as doublet bands, suggesting that the latter arose from multiple DNA-bound cohesins. All experiments were repeated, and representative examples are shown. See also Figure S3 for further characterization of the 6C cohesin crosslinking experiment.

We loaded 6C cohesin onto the 3.2 kb plasmid and used it as the template for DNA replication. Following replication, we added BMOE to covalently circularize cohesin. Next, we denatured all proteins by heating in the presence of 1% SDS. Following denaturation, we diluted the sample to reduce the SDS concentration and enable cohesin immunoprecipitation. Finally, we eluted cohesin-bound replication products from the antibody beads (Figure 2A). Separation of these eluates by agarose gel electrophoresis, following deproteinization by Proteinase K treatment, revealed retention of replication products by 6C cohesin treated with BMOE, but not by 5C cohesin, or by 6C cohesin in the absence of crosslinker. Alongside, we confirmed that the cohesin immunoprecipitation efficiency was consistent between the three experimental conditions (Figure S3D). This result demonstrates that cohesin topologically embraced *in vitro*-synthesized DNA replication products.

We next repeated the above experiment, this time analyzing the cohesin-bound DNA replication products without Proteinase K treatment. If sister chromatid cohesion was established during DNA replication, the denatured but covalently closed cohesin rings should link both replication products. Agarose gel electrophoresis of this eluate showed two prominent groups of products: faster migrating products close to where we expect monomer 3.2 kb circles (Figure 2B, **F** products) and slower migrating products at approximately double that size (**S** products). To create an expectation for where two conjoined replication products would migrate, we performed a replication reaction from which we omitted Top II. Without Top II, products around the size of **S** products increased in intensity, at the expense of **F** products, consistent with the interpretation that the fast- and slow-migrating bands correspond to monomer and interlinked dimer replication products.

To address whether the **S** band contained pairs of replication products that were held together by cohesin rings, rather than being catenanes, we separated 6C cohesin-associated replication products by two-dimensional (2D) gel electrophoresis. The first dimension was conducted as before, separating the fast- and slow-migrating populations on a native agarose gel. This gel lane was excised and applied to a perpendicular gel where the products migrated through a Proteinase-K-containing stripe before separation along an SDS-containing agarose gel (Figure 2C). Any protein-dependent sister chromatid cohesion should be destroyed by Proteinase K before separation in the second dimension. While most of the replication products showed comparable migration in the first and second dimensions, forming a diagonal, a distinct fraction of the **S** products was converted to **F** products following Proteinase K treatment. This observation suggests that part of the 6C cohesin-associated replication products were protein-dependent dimers, i.e., they were most likely held together by the 6C cohesin ring.

To test whether protein-dependent plasmid dimers were indeed held together by cohesin rings, we inserted a TEV protease recognition sequence into the Scc1 subunit of the 6C cohesin ring (Scc1 TEV, Figure S3E).^39^ We then repeated the replication-coupled cohesion establishment experiment using 6C cohesin containing either wild-type Scc1 or Scc1 TEV. Following crosslinking and denaturation, we treated the cohesin immuno-precipitates with or without TEV protease before analyzing the samples by 2D gel electrophoresis (Figure 2D). Protein-dependent dimers were no longer observed following TEV protease treatment of Scc1 TEV-associated replication products but persisted in mock-treated samples or if TEV protease was used to treat replication products linked to wild-type Scc1. This experiment reveals that *in vitro* replication of a cohesin-bound DNA template results in dimer replication products that are topologically held together by cohesin rings.

### The specificity of *in vitro* sister chromatid cohesion establishment

**Figure 3:**
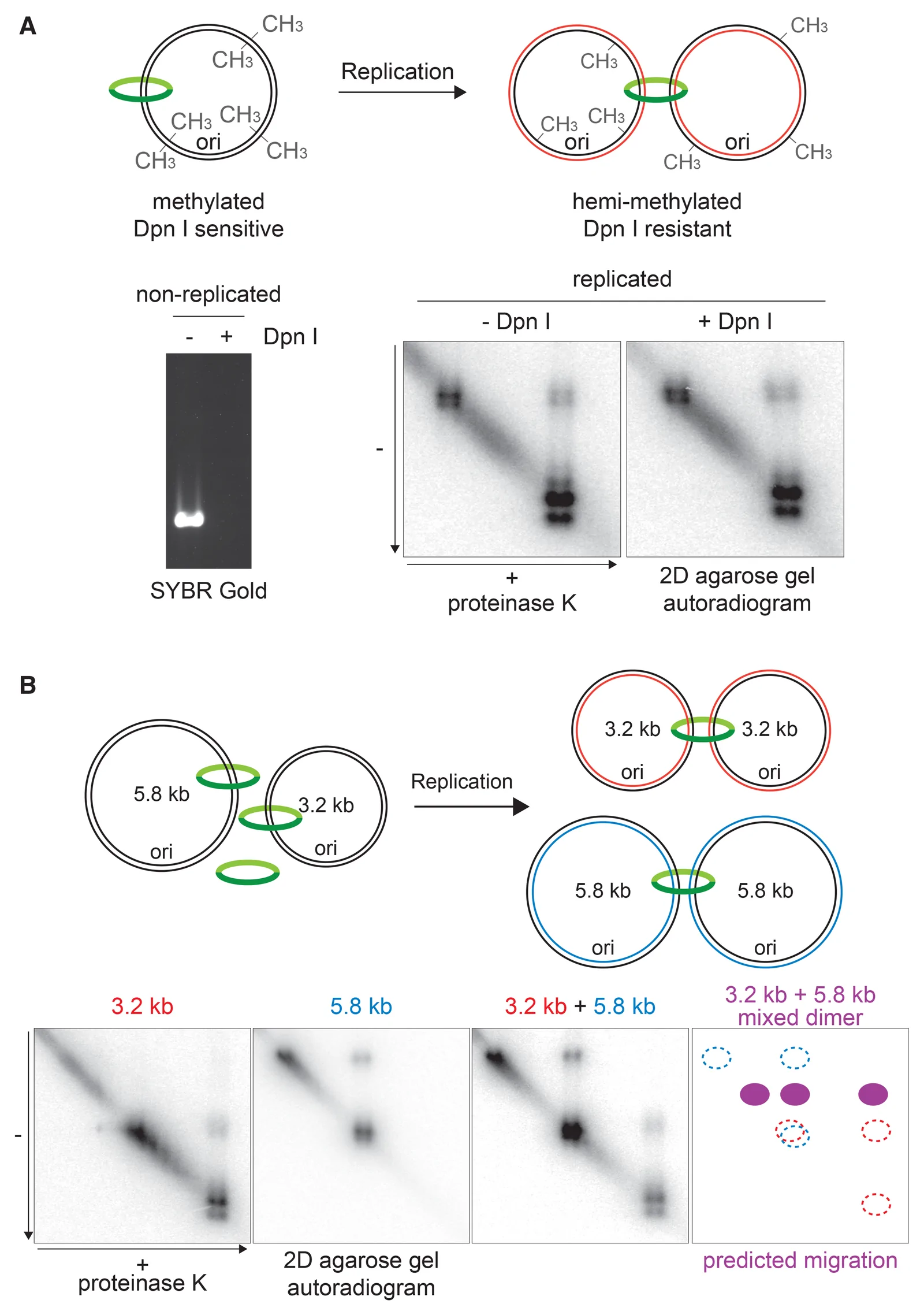
The specificity of sister chromatid cohesion establishment (A) Replication schematic of a methylated DNA template and experiments to test the Dpn I restriction enzyme sensitivity of the template DNA, as well as of 6C cohesin-associated replication products analyzed by 2D gel electrophoresis. (B) Schematic of a replication reaction containing two template DNAs of different sizes and an experiment to analyze 6C cohesin-associated replication products of reactions containing either, or both, templates using 2D gel electrophoresis. The expected band positions produced by mixed dimers, i.e., migrating between the two homodimer sizes in the first dimension and resolving into both monomer sizes in the second dimension, are indicated in a schematic. The experiments presented in this figure were repeated, and representative examples are shown. See also Figure S4 for one-dimensional agarose gel electrophoresis of replication products produced in the presence of either, or both, templates, as well as for confirmation of *in vitro* replication-coupled cohesin acetylation.

Next, we needed to establish whether the two DNAs held together by cohesin rings were indeed the two sister chromatids produced at a replication fork, rather than two unrelated DNAs that cohesin might have embraced. Therefore, we asked whether both DNAs in cohesin-linked dimers were replication products, or whether one of the two might be an unreplicated template DNA circle that is present in excess in the replication reaction. To investigate this possibility, we took advantage of the Dam methylation status of the template DNA isolated from *E. coli*. Methylation of both DNA strands makes the template a target for the Dpn I restriction endonuclease. Following *in vitro* replication, the two sister chromatids are each hemi-methylated and thereby turn resistant to Dpn I treatment. We confirmed that our template DNA, containing 16 Dpn I recognition sites, was efficiently degraded following Dpn I treatment (Figure 3A). On the other hand, similar Dpn I treatment of the dimer replication products, associated with 6C cohesin, showed that these were resistant to Dpn I treatment. This observation shows that cohesin-dependent DNA dimers consist of two newly synthesized replication products.

Cohesin must specifically establish interactions between the two sister chromatids that emanate from a replication fork, not between any newly replicated sequences. To test the specificity of *in vitro* sister chromatid cohesion establishment, we performed a replication reaction that contained two DNA circles of different sizes. We loaded 6C cohesin onto the 3.2 kb, as well as onto a 5.8 kb replication origin-containing plasmid. We then mixed the two templates before MCM loading, activation, and DNA replication. Both templates in this mixed reaction were replicated with comparable efficiencies (Figure S4A). We then separated 6C cohesin-associated replication products by 2D gel electrophoresis. This analysis revealed two types of protein-mediated DNA dimers. The two types corresponded to bands observed in reactions that contained either only the 3.2 kb or only the 5.8 kb template (Figure 3B). We did not detect a signal at positions where a mixed 3.2–5.8 kb dimer would be expected to migrate. From these observations, we conclude that cohesin establishes topological interactions specifically between the two sister chromatids that are produced during *in vitro* DNA replication.

### Replication-coupled cohesin acetylation

In addition to co-entrapment of replicated DNAs, successful sister chromatid cohesion establishment requires acetylation of cohesin’s Smc3 subunit by the Eco1 acetyl transferase.^25,26^ Immunoblotting of replication products using an acetyl-Smc3-specific antibody revealed that Smc3 indeed became acetylated during *in vitro* cohesion establishment (Figure S4B). We have previously characterized the requirements for cohesin acetylation, which include a combination of replication and cohesion factors, as well as transient DNA structures, flaps, or nicks that form during Okazaki fragment processing.^31^ Except for confirmation that cohesin acetylation takes place as part of our *in vitro* reconstituted sister chromatid cohesion establishment, we will here not further investigate the acetylation reaction.

### Cohesin often embraces only one of the two replication products

In addition to cohesin-mediated DNA dimers changing from slow to fast migration during 2D gel electrophoresis, 6C cohesin also recovered replication products that did not change migration following Proteinase K or TEV protease treatment (Figures 2C and 2D). Proteolysis only partly resolved the slower migrating **S** band toward fast **F** migration, suggesting that some of the dimer replication products were connected by protein-independent links, most likely by catenation. Catenanes form as the result of replication termination, and their resolution by Top II remains incomplete, as this enzyme not only removes but also introduces intertwines. Catenanes are additionally protected from resolution if the two DNAs are connected by cohesin.^40,41^ It is therefore likely that diagonal **S**-band products include DNA dimers connected by both cohesin and DNA catenation.

Strikingly, a fraction of replication products retrieved by 6C cohesin were monomer circles, migrating in the **F** position in both dimensions during 2D gel electrophoresis (Figures 2C and 2D). How might monomer replication products become topologically entrapped by cohesin? One possible explanation is that *in vitro* established sister chromatid cohesion is unstable over time, leaving some cohesins embracing only one of the two sisters at the time of crosslinking. To investigate this possibility, we performed a time course experiment. DNA replication is largely complete after 30 min (Figure S5A). We then continued the incubation for an additional 30 or 60 min before 6C cohesin crosslinking. 2D gel electrophoresis showed that the fraction of protein-mediated dimers remained during this extended incubation (Figure S5B). This outcome demonstrates that cohesin-mediated DNA dimers, once formed, are stable.

The second possible explanation for cohesin-bound monomer replication products is that they have arisen during DNA replication. This scenario implies that *in vitro* DNA replication results in a proportion of cohesins that entrap only one of the two replication products. This outcome is consistent with our previous observation that a smaller fraction of duplicated replication products was recovered by cohesin when compared with the input DNA (Figure 1B). We will explore the possible origin and implications of cohesin-bound monomer replication products in the discussion.

### Cohesion establishment factors and *in vitro* sister chromatid cohesion establishment

**Figure 4:**
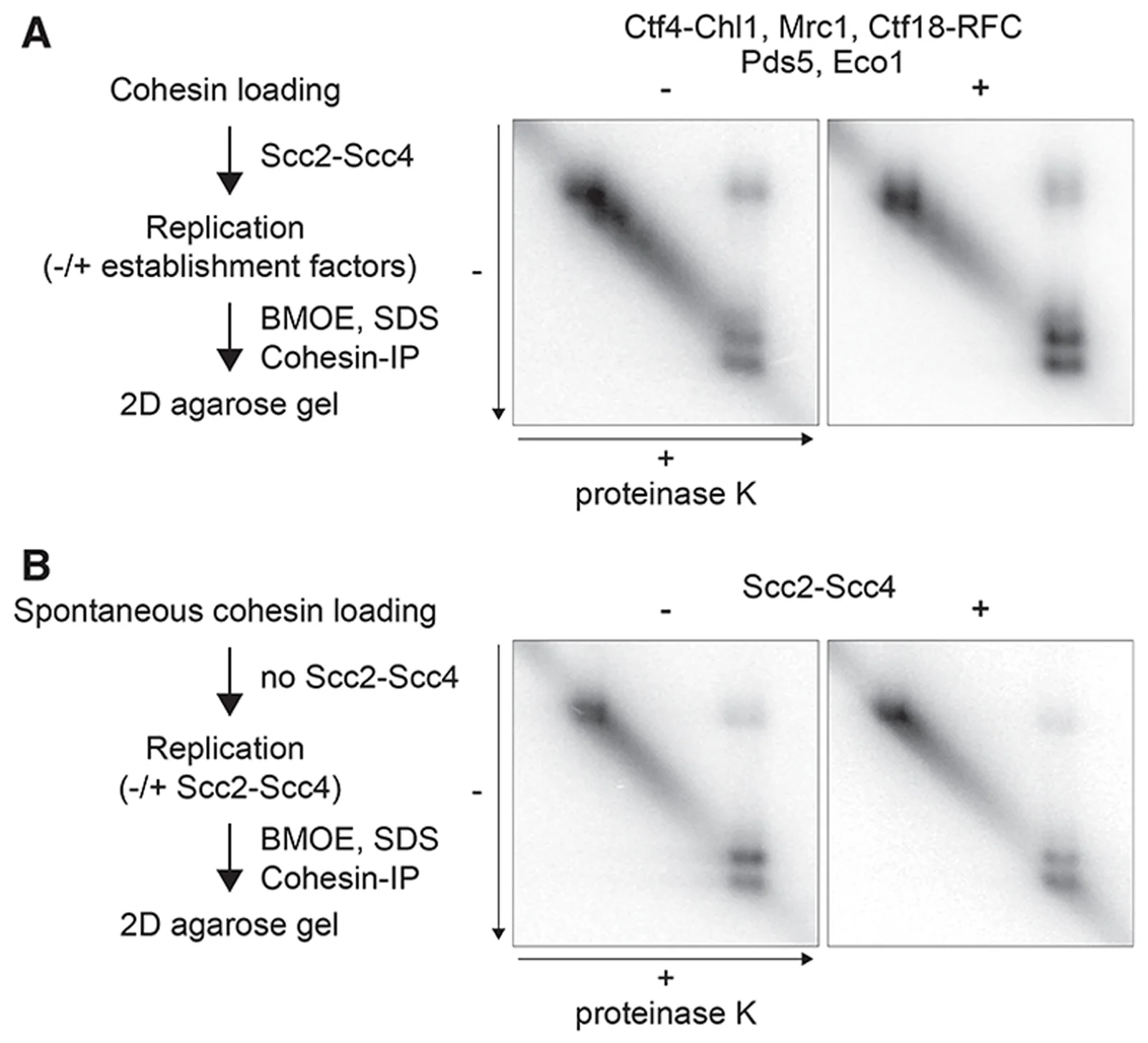
Contribution of cohesion establishment factors and cohesin loader to sister chromatid cohesion establishment (A) 6C cohesin-bound templates were replicated in the presence or absence of the indicated cohesion establishment factors. Cohesion establishment was assessed following crosslinking, denaturation, and cohesin IP by 2D gel electrophoresis. The experiment was repeated three times, and a representative example is shown. (B) 6C cohesin was loaded onto the template DNA in a reaction without the Scc2–Scc4 cohesin loader, then replication was conducted in the presence or absence of the cohesin loader, and cohesion establishment was monitored as in (A). The experiment was repeated, and a representative example is shown. See also Figure S5 for a time course analysis to probe the stability of *in vitro* established sister chromatid cohesion.

We next explored the contribution of known cohesion establishment factors to *in vitro* sister chromatid cohesion establishment. To do so, we replicated a 6C cohesin-bound plasmid DNA template, side by side, in the presence or absence of Ctf4-Chl1, Mrc1, Ctf18-RFC, Eco1, and Pds5. 2D gel electrophoresis revealed that cohesin-mediated dimer products were generated both in the presence and absence of cohesion establishment factors (Figure 4A). This observation suggests that *in vitro* cohesion establishment proceeds independently of replisome-associated cohesion establishment factors. The role of these factors therefore pertains either to cohesion establishment in a more complex *in vivo* setting where cohesion establishment must be coordinated with histone inheritance,^42^ or the role of cohesion establishment factors might pertain to promoting the cohesin acetylation reaction in which they are also involved.^24,32^ We were unable to test the role of Tof1-Csm3 in *in vitro* cohesion establishment, as DNA replication was too inefficient in their absence.^34^ Though we note that the contribution of Tof1-Csm3 to cohesion establishment is thought to overlap with that of Ctf4-Chl1.^23^

In addition to its role in cohesin loading onto chromosomes, the Scc2–Scc4 cohesin loader contributes to cohesion establishment during the S phase.^10,31,43^ To investigate a possible Scc2–Scc4 role during *in vitro* cohesion establishment, we took advantage of the fact that *in vitro* cohesin loading proceeds, albeit at a reduced rate, without the cohesin loader.^6,35^ We therefore increased the 6C cohesin loading time without a loader, then used this DNA template with 6C cohesin in a DNA replication reaction. DNA replication was then performed either without or with added cohesin loader. 2D gel electrophoresis of cohesin-bound replication products revealed cohesion establishment both with or without the cohesin loader (Figure 4B). Taken together, these observations reveal that the cohesion establishment that we observe during *in vitro* DNA replication occurs independently of cohesion establishment factors and independently of the cohesin loader.

### Replication-coupled cohesin loading

**Figure 5:**
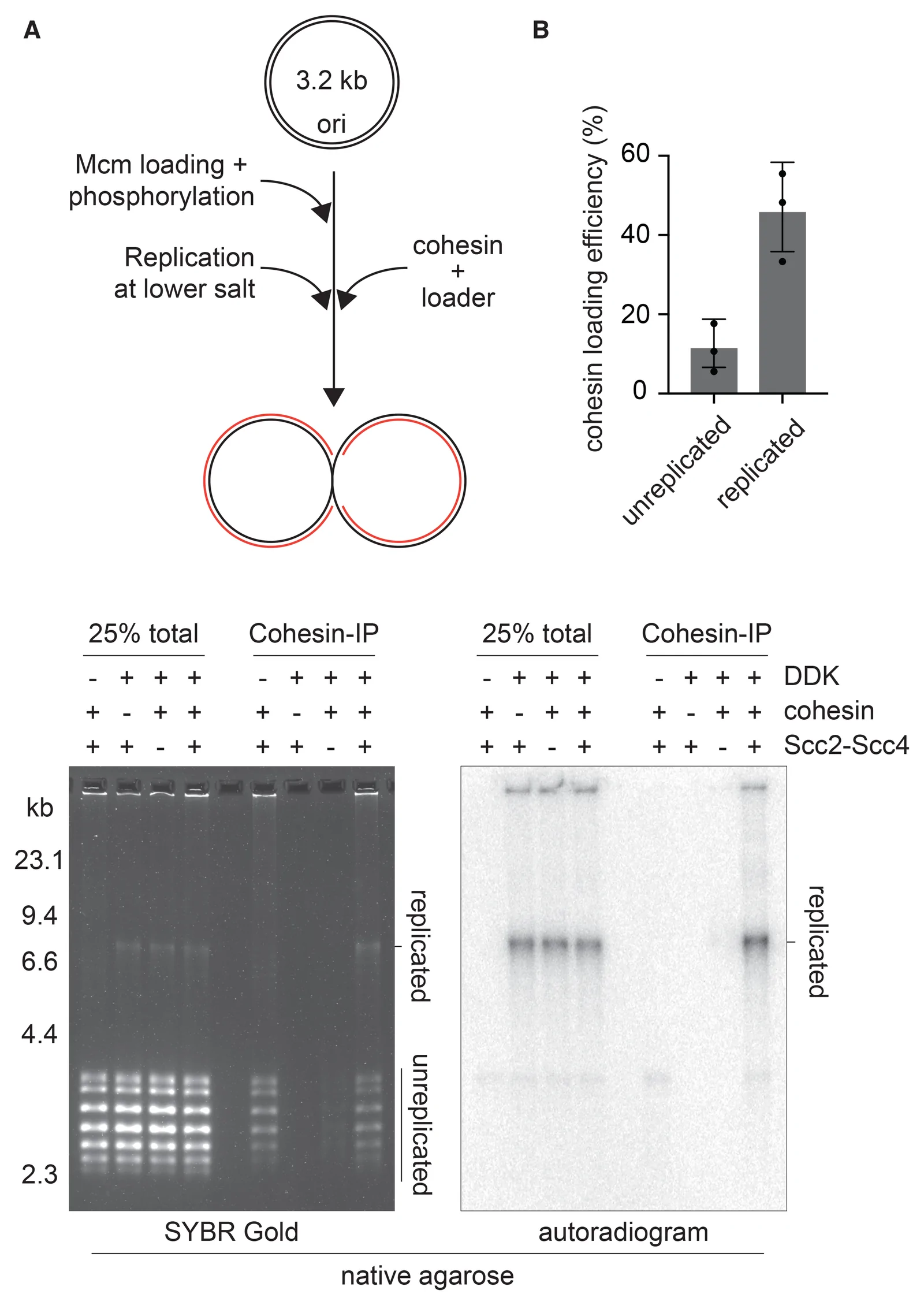
Replication-coupled cohesin loading (A) Schematic of a replication reaction during which cohesin and the cohesin loader were added at the time of DNA replication. (B) Representative gel electrophoretic analysis of the total DNA following the replication incubation and of the cohesin-bound DNA recovered following cohesin IP. DNA was visualized by SYBR Gold, and replication products by [α-^32^P]dCTP autoradiography. Top II was omitted from this experiment so that late replication intermediates accumulate that migrate distinctly slower than the template DNA. Cohesin loading onto unreplicated and replicated DNA was quantified in three independent repeat experiments (black circles). The means (bars) and standard deviations (error bars) are indicated. See also Figure S6 for a characterization of the role of cohesion establishment factors in replication-coupled cohesin loading.

Our results so far demonstrate cohesion establishment by cohesin that was bound to DNA before DNA replication. We asked whether soluble cohesin complexes that are present during DNA replication also contribute to sister chromatid cohesion establishment. In Figure 1A, we saw very little cohesin recruitment during DNA replication, at least if replication was carried out at relatively high ionic strength conditions that impede *in vitro* cohesin loading. We therefore repeated DNA replication reactions at a lower salt concentration of 100 mM potassium glutamate and using a cohesin-free template DNA for MCM loading and activation. Cohesin and its loader were then added at the time of origin firing and replication initiation (Figure 5A). After the replication incubation, we assessed cohesin loading. Quantification of cohesin-bound DNA revealed that almost 50% of replication products were bound by cohesin (Figure 5B). This level of recovery was substantially greater than the recovery of template DNA that remained unreplicated in the same incubation, only approximately 10% of which was bound by cohesin. Cohesin loading onto replicated DNA strictly depended on the cohesin loader (Figure 5A). These findings reveal that DNA replication prompts cohesin association with replication products to a level that goes well beyond cohesin’s natural tendency to load onto non-replicating DNA. In fact, the degree of replication-coupled cohesin loading exceeded any previously observed *in vitro* cohesin loading efficiencies.^6,35^

### Cohesion establishment by replication-coupled cohesin loading

**Figure 6:**
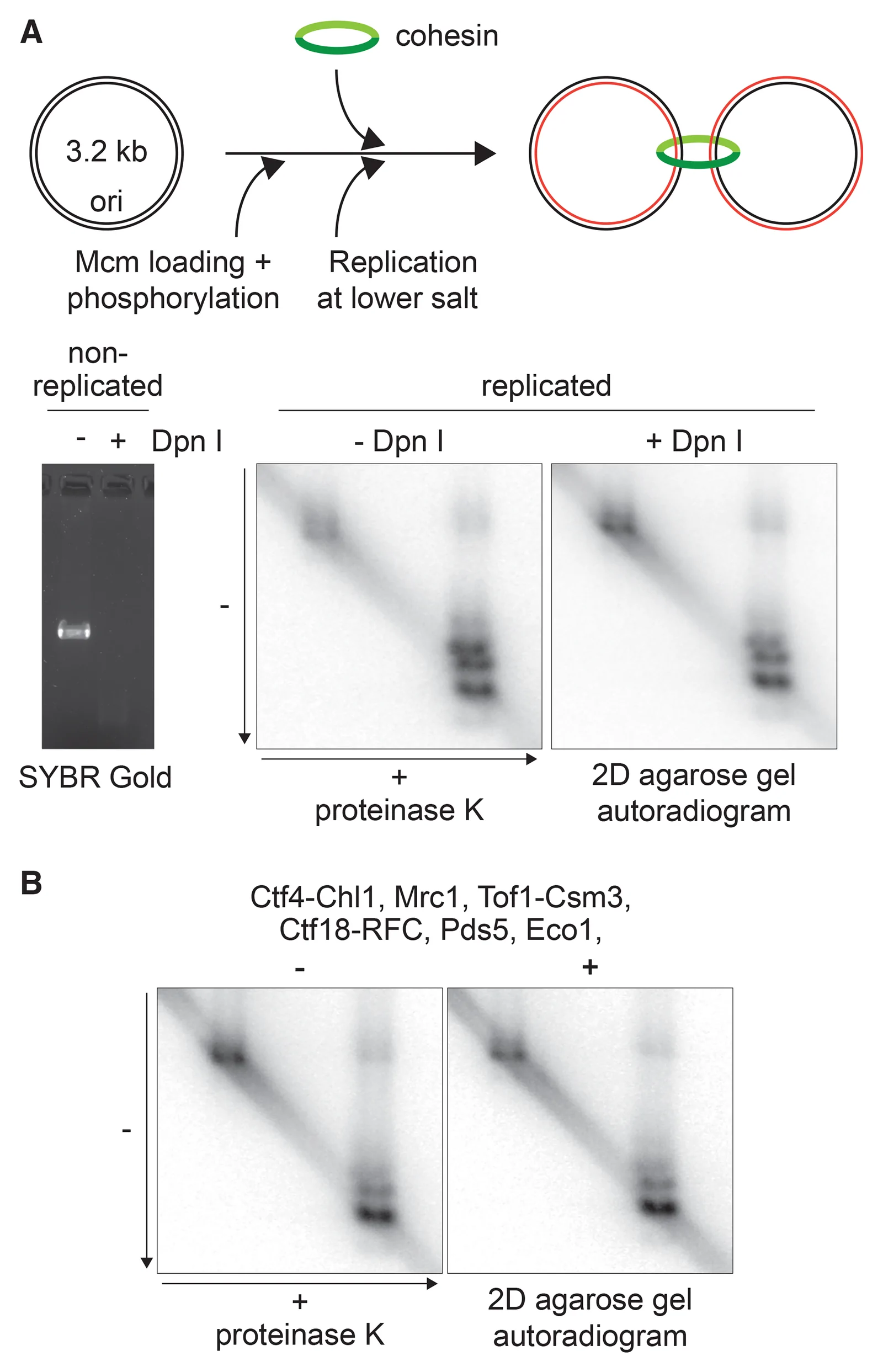
Cohesion establishment during replication-coupled cohesin loading (A) Schematic of a complete replication reaction during which 6C cohesin and the cohesin loader were added at the time of DNA replication. The Dpn I sensitivity of the template DNA was analyzed, as well as that of the replication products that were analyzed by 2D gel electrophoresis following cohesin crosslinking, denaturation, and cohesin IP. This experiment was performed once. (B) As in (A), but DNA replication was conducted in the presence or absence of the indicated cohesion establishment factors. The experiment was repeated, and a representative example is shown.

We lastly wanted to know whether replication-coupled cohesin loading results in the establishment of sister chromatid cohesion. To answer this question, we added 6C cohesin together with the cohesin loader during DNA replication. Following completion of DNA replication, we added BMOE to covalently close cohesin rings, performed SDS denaturation, and immunopurified topologically entrapped replication products. Analysis by 2D gel electrophoresis revealed the generation of protein-dependent plasmid dimers, suggesting that replication-coupled cohesin loading resulted in the establishment of sister chromatid cohesion (Figure 6A). The dimer species, but not the input template, was resistant to Dpn I treatment, confirming that they consisted of two replication products. Therefore, sister chromatid cohesion can be established by cohesin that is recruited to DNA during ongoing DNA replication.

In addition to plasmid dimers, 6C cohesin topologically associated with monomer replication products (Figure 6A). This observation suggests, as during cohesion establishment using pre-loaded cohesin, that a frequent outcome of cohesin loading during DNA replication is cohesin entrapment of only one of the two replication products.

We lastly tested whether cohesion establishment by replication-coupled cohesin loading depended on replication fork-associated cohesion establishment factors. The degree of replication-coupled cohesin loading remained unchanged in reactions lacking Ctf4-Chl1, PCNA, Mrc1, or Tof1-Csm3 (Figure S6A). Similarly, protein-dependent dimer products formed both in reactions containing or lacking Ctf4-Chl1, Mrc1, Tof1-Csm3, Ctf18-RFC, Pds5, and Eco1 (Figure 6B). Sister chromatid cohesion establishment by co-entrapment of the two DNA replication products is therefore a process that can occur independently of replisome-associated cohesion establishment factors.

Murayama et al.^36^ reported that the Chl1 helicase promotes replication-coupled cohesin loading. As we did not observe a similar Chl1 contribution, we confirmed that our purified Chl1 was enzymatically active (Figure S6B). While the reason for the divergent observations thus remains to be explored, we can conclude that DNA replication promotes cohesin loading in both Chl1-independent (our experiments) and Chl1-dependent^36^ ways and that replication-coupled cohesin loading results in topological entrapment of either one or both sister chromatids.

## Discussion

Here, we report the *in vitro* reconstitution of sister chromatid cohesion establishment during DNA replication. Biochemical reconstitution remains a crucial test of our understanding of cellular events. While genetic approaches have identified the many components of the sister chromatid cohesion machinery, we have now confirmed that we know of all the essential parts that are required to ensure that replicated genome copies, synthesized during S phase, remain topologically connected to one another by the cohesin ring. At the same time, our biochemical investigations have begun to reveal unexpected insight into the molecular events that lead to sister chromatid cohesion.

### Replication encounters with pre-loaded cohesin

Cohesin that is loaded onto DNA before the onset of DNA replication results in topological entrapment of pairs of *in vitro* replicated sister chromatids. Cohesion establishment was equally efficient if replication was performed by a stripped-down replisome, encompassing the bare essentials to carry out DNA synthesis and lacking many of the components that have been identified as *in vivo* sister chromatid cohesion establishment factors. The cohesin loader was also dispensable for this type of sister chromatid cohesion establishment. These observations reveal an intimate relationship between DNA replication and sister chromatid cohesion. As soon as the ring-like cohesin architecture became known, the possibility emerged that replisomes might simply pass through these rings to leave the replication products trapped inside.^12,13^ Recent single-molecule observations of real-time replisome-cohesin encounters have indeed depicted the replisome apparently slipping through cohesin rings to establish sister chromatid cohesion.^44^ It is likely that the same occurs in our bulk biochemical experiments, explaining one of the pathways by which cohesion is established when replication meets cohesin.

In addition to establishing cohesion between both replicated DNAs, we found that cohesin often entrapped only one of the two replication products. Following *in vivo* plasmid replication, cohesin has similarly been seen to entrap either both or only one of the replication products.^7^ This parallel suggests that cohesin-bound monomer replication products are not an *in vitro* artifact but likely part of cohesin’s physiological behavior. While entrapped monomer replication products were a prominent outcome of our reactions, it is not immediately obvious how cohesin transitions from entrapping the template DNA to entrapping only one of the two replication products. Cohesin would have to come off the DNA and then capture only one of the two sister chromatids when getting back on. However, the incidence of single capture events was unchanged in reactions from which we omitted the cohesin loader, suggesting that cohesin unloading and reloading was not part of the transfer mechanism. An alternative explanation for monomer entrapment is that the cohesin ring ruptured during replisome passage and closed again around only one of the two sisters. The weakest cohesin ring interface at the hinge is known to break at ~20 pN, a force in the range of that produced by advancing replisomes.^11^ Single capture products could therefore be an unwanted but unavoidable outcome of replisome-cohesin encounters, with only a fraction of cohesins surviving intact to reach productive sister chromatid cohesion.

Could single capture events be salvaged and converted into sister chromatid cohesion? A hint for this possibility comes from *in vivo* analyses of how the cohesin loader contributes to sister chromatid cohesion establishment.^10,13,43^ Like in our *in vitro* reactions, a basal level of sister chromatid cohesion is achieved independently of the cohesin loader. However, the presence of the cohesin loader during DNA replication results in much more robust *in vivo* sister chromatid cohesion establishment,^10^ as well as in a greater proportion of entrapped plasmid dimers over monomers.^7^ Whether the cohesin loader acts in cohesion establishment by converting single to double capture events will be important to explore, as well as why this reaction might have been poorly recapitulated in our *in vitro* replication reactions (the salt sensitivity of the *in vitro* cohesin loading reaction might have been a confounding factor).

### Replication-coupled cohesin loading and cohesion establishment

An additional reason to consider single-to-double capture transitions as a mechanism for cohesion establishment comes from observations with cohesin that is newly recruited during DNA replication. In this scenario, cohesin is again seen embracing either only one of the sister chromatids or co-entrapping both. This observation is easiest explained by a sequential DNA-DNA capture mechanism, with a first loading event followed by a second. The reaction could be akin to previous *in vitro* observations in which cohesin sequentially captures a double-stranded DNA, followed by a single-stranded DNA.^10^ This substrate geometry is reflected at the replication fork, where the dsDNA leading strand products lie juxtaposed to the unwound single-stranded DNA (ssDNA) lagging strand template. In the future, it will be important to explore whether the first DNA capture event (or retention of previously loaded cohesin) more often occurs on one than the other, the leading or the lagging strand. Such information could provide clues as to how a sequential capture mechanism operates at replication forks.

Replication-coupled *de novo* cohesin loading has been hypothesized to be the role of the Mrc1 and Ctf18-RFC cohesion establishment factors.^7,43^ However, we find that Mrc1 and Ctf18-RFC are not in fact required for replication-coupled cohesin loading, a conclusion corroborated by Murayama et al.^36^ How Mrc1, Ctf18-RFC, and other cohesion establishment factors contribute to building sister chromatid cohesion therefore remains to be understood. *In vivo*, the replisome must coordinate cohesin transfer and DNA capture reactions with histone transfer and redeposition. DNA that is wrapped around histones is no longer a substrate for cohesin loading.^45^ The Tof1 and Mrc1 cohesion establishment factors have known roles in histone transfer,^46,47^ while Ctf18-RFC-loaded PCNA might coordinate *de novo* histone deposition.^48^ It is therefore possible that the role of cohesion establishment factors chiefly lies in directing histone deposition, such that cohesin retains the required DNA access for cohesion establishment. Alternatively, or additionally, the role of cohesion establishment factors could lie in linking sister chromatid co-entrapment and the cohesin acetylation reaction.^24,31^

### More than one way to establish sister chromatid cohesion

A prior publication by Murayama et al.^36^ investigated biochemically reconstituted replisome-cohesin encounters. The study similarly reported that budding yeast cohesin loaded onto DNA before replication remains DNA bound throughout complete DNA synthesis and that known cohesion establishment factors are dispensable for this process. Based on an increased proportion of catenated replication products in the presence of cohesin, the authors suggested that cohesin established sister chromatid cohesion. Our study substantiates these results and goes further by demonstrating that cohesin rings remain topologically DNA bound during DNA replication and physically encircle both replication products. The use of 6C cohesin to characterize these cohesed replication products led us to realize that cohesin does not always successfully entrap both sisters but often entraps only one of the two replication products. As replication-coupled cohesin loading results in a similar pattern of singly or doubly entrapped replication products, we speculate that single entrapment is an intermediate on the way to establishing cohesion between both replicated DNAs. Taken together, our results provide evidence for three ways of establishing sister chromatid cohesion: (1) replication fork passage through cohesin rings^44^; (2) cohesin ring rupture of preloaded cohesin and embrace of one replication product, with a possible salvage pathway to entrapping both; and (3) sequential replication-coupled cohesin loading onto one and then the other replication product.

### Limitations of the study

Our *in vitro* studies have begun to unravel the molecular events that take place during sister chromatid cohesion establishment at DNA replication forks. In the future, it will be important to perform cohesion establishment experiments using histone-bound substrates to investigate the roles of cohesion establishment factors in the context of chromatin replication. Furthermore, it will be important to develop methods in living cells that can discern the different cohesion establishment scenarios that we observed *in vitro*. Such approaches will allow asking whether, and to what extent, these different pathways are operational *in vivo*. It will also be interesting to explore whether different pathways might be tailored to distinct chromatin environments. Together, these studies will further our molecular understanding of the chromosome replication and segregation cycle.

## Resource Availability

### Lead contact

Further information and requests for resources and reagents should be directed to and will be fulfilled by the lead contact, Frank Uhlmann (frank.uhlmann@crick.ac.uk).

### Materials availability

All unique reagents generated in this study will be made available upon reasonable request without restrictions.

### Data and code availability

- All data reported in this paper will be shared by the lead contact upon request. Original gel images have been made available on the Mendeley repository, where they can be accessed at https://data.mendeley.com/datasets/jykrkr88pj/2.
- This paper does not report original code.
- Any additional information required to reanalyze the data reported in this paper is available from the lead contact upon request.

## Star★Methods

### Key Resources Table

**Table:** 

REAGENT or RESOURCE	SOURCE	IDENTIFIER
Antibodies		
Mouse monoclonal anti-V5(Pk)	Bio-Rad	Cat# MCA1360; RRID:AB_322378
Monoclonal ANTI-FLAG® M2 antibody produced in mouse	Sigma-Aldrich	Cat# F3165; RRID:AB_259529
Goat anti-myc tag antibody, affinity purified	Bethyl Laboratories	Cat# A190-104A; RRID:AB_66864
Mouse monoclonal anti-acetylated Smc3	Gift from the Shirahige laboratory	N/A
Chemicals, peptides, and recombinant proteins		
Rabbit IgG-Agarose	Merck	Cat# A2909
TALON Metal Affinity Resin	Clonetech	Cat# 635502
HiTrap Heparin HP 1ml	Cytiva	Cat# 17-0407-01
HiTrap SP HP 1ml	Cytiva	Cat# 29-0513-24
Superose 6 Increase 10/300 GL	Cytiva	Cat# 29-0915-96
Superdex 200 Increase 10/300 GL	Cytiva	Cat# 28-9909-44
Superdex 75 10/300 GL	Cytiva	Cat# 17-5174-01
cOmplete, EDTA-free Protease Inhibitor Cocktail	Merck	Cat# 04693132001
Pefabloc SC	Roche	Cat# 11429876001
SYBR Gold nucleic acid gel stain	ThermoFisher	Cat# S11494
Acetyl coenzyme A lithium salt	Merck	Cat# A2181
T5 Exonuclease	NEB	Cat # M0663
ATP	ThermoFisher	Cat# R0441
TCEP	Fluorochem Limited	Cat# M02624
Proteinase K	Merck	Cat# 107393
Calmodulin Affinity Resin	Agilent	Cat# 214303
ANTI-FLAG® M2 Affinity Gel	Merck	Cat# A2220
SIGMAFAST™ Protease Inhibitor Cocktail Tablets, EDTA-Free	Sigma-Aldrich	Cat# S8830
QIAGEN Plasmid Mega Kit	QIAGEN	Cat# 12181
5 mL, Open-Top Thinwall Ultra-Clear Tube,	BECKMAN COUTER	Cat# 344057
[α-^32^P]dCTP, 3000 Ci/mmol, 20 mCi/ml	HARTMANN ANALYTIC	Cat# FP-205H
Amersham MicroSpin G-50 Columns	Cytiva	Cat# 27533001
BMOE (bis-maleimidoethane)	Thermo Fisher SCIENTIFC	Cat# 22323
Dpn I	NEB	Cat# R0176
Nb.BssSI	NEB	Cat# R0681
Dynabeads™ Protein G for Immunoprecipitation	Invitrogen	Cat# 10004D
Experimental models: Yeast strains		
Most yeast strains were previously described. The following additional strains were used in this study.	Posse et al.^49^	N/A
*MAT**a** ade2-1 can1-100 ura3 trp1 GAL* *psi+ pep4Δ::HIS3 wpl1Δ::LEU2 eco1Δ::KAN^MX6^pMM40(pRSII402-pGAL-GAL4+SMC1 G22C, K639C-Pk_3_) pMM38(YIplac204-pGAL-SMC3 E570C, S1043C+SCC1 A547C-3C-ProtA_2_) pMM92 (YIplac211-pGAL-Scc3-FLAG)*	This study	Y6748
*MAT**a** ade2-1 can1-100 ura3 trp1 GAL* *psi+ pep4Δ::HIS3 wpl1Δ::LEU2 eco1Δ::KAN^MX6^* *pMM40(pRSII402-pGAL-GAL4+SMC 1 G22C-Pk_3_) pMM38(YIplac204-pGAL-SMC3 E570C, S1043C+SCC1 A547C-3C-ProtA_2_) pMM92(YIplac211-pGAL-Scc3-FLAG)*	This study	Y6842
*MAT**a** ade2-1 can1-100 ura3 trp1 leu2 his3 GAL psi+ pep4Δ::Hyg^MX6^ pMM128(pRSII402-pGAL-GAL4+ FLAG-HA-CHL1)*	This study	Y6943
*MAT**a** ade2-1 can1-100 ura3 trp1 leu2 his3 GAL psi+ pep4Δ::Hyg^MX6^ pMM122(pRSII402-pGAL-GAL4+Chorella virus Top II-FLAG)*	This study	Y6850
Recombinant DNA		
pBluescript harboring *ARS1* and approximately 0.2 kb surrounding region (total size 3.2 kb)	On et al.^50^	N/A
pBluescript harboring *ARS1* and approximately 2.8 kb surrounding region (total size 5.8 kb)	Yeeles et al.^33^	N/A

### Experimental Model and Study Participant Details

#### Yeast strains

Budding yeast *S. cerevisiae* strains used in this study are listed in key resources table. Cells were grown in YP medium containing 2% glucose (YPD), 2% raffinose, or 2% raffinose + 2% galactose as carbon source at 30 °C. The conditions used for induction of protein expression are detailed under the respective subsections of the methods details.

### Method Details

#### Protein expression and purification

Budding yeast replication proteins (ORC, Cdt1-Mcm2-7, Cdc6, DDK, S-CDK, RPA, Dpb11, Cdc45, GINS, Top I, Top II, Ctf4, Pol ε, Mcm10, Tof1-Csm3, Mrc1, RFC, PCNA, Pol δ, Fen1, Cdc9, Pol α, Sld2, Sld3-Sld7),^49^ Pif1 and Dna2,^51^ Eco1, Ctf18-RFC, Pds5,^31^ and cohesin and the Scc2-Scc4 cohesin loader complexes^35^ were purified following published protocols.

#### 6C Cohesin purification

Budding yeast cells overexpressing Smc1(G22C, K639C)-Pk, Smc3(E570C, S1043C), Scc1(A547C)-Protein A, and Scc3-FLAG were grown in YP medium containing 2% raffinose as the carbon source to an optical density of 1.0 at 30 °C. 2% galactose was then added to the culture to induce protein expression, and cells were further grown for 2 hours. Cells were collected by centrifugation, washed with deionized water, and suspended in cohesin buffer (50 mM HEPES-KOH pH 7.5, 20% glycerol) containing 300 mM NaCl, 0.5 mM TCEP, 2 mM MgCl_2_, 0.5 mM Pefabloc, as well as cOmplete-EDTA protease inhibitor cocktail. The cell suspension was frozen in liquid nitrogen, then cells were broken in a cryogenic freezer mill. The cell powder was thawed on ice, and further cohesin buffer containing 300 mM NaCl, 0.5 mM TCEP, 0.5 mM MgCl_2_ and protease inhibitors was added. The lysate was clarified by centrifugation at 20,000 x g for 1 hour. RNase A and benzonase were added to a final concentration of 0.3 μg/ml and 1.25 U/ml, respectively, to the clarified lysate. The lysates were transferred to pre-equilibrated Rabbit IgG agarose resin and incubated for 2 hours. The resin was washed with cohesin buffer containing 300 mM NaCl, 0.5mM TCEP, and 2 mM MgCl_2_, then incubated in cohesin buffer containing 300 mM NaCl, 0.5 mM TCEP, 10 mM MgCl_2_ and 1 mM ATP for 15 minutes. The resin was washed again with cohesin buffer containing 300 mM NaCl, 0.5 mM TCEP, and 2 mM MgCl_2_, then incubated overnight in the same buffer containing 10 μg/ml PreScission protease. The eluate was collected, and loaded onto a HiTrap Heparin column, equilibrated with cohesin buffer containing 300 mM NaCl and 2.5 mM TCEP. The column was developed with a linear gradient from 300 mM to 1 M NaCl in cohesin buffer containing 2.5 mM TCEP. The peak fractions were pooled and loaded onto a Superose 6 Increase 10/300 GL gel filtration column that was equilibrated and developed with cohesin 6C gel filtration buffer (20 mM Tris-HCl pH 7.5, 150 mM NaCl, 10% Glycerol, 2.5 mM TCEP). The peak fractions were combined and concentrated by ultrafiltration.

#### 5C and 6C cohesin TEV purification

Cysteine pairs to covalently close the cohesin ring were introduced as described.^52^ Budding yeast cells overexpressing Smc1(G22C, K639C)-Pk or Smc1(G22C)-Pk, Smc3(E570C, S1043C), Scc1(A547C)-CBP harboring a TEV protease site (ENLYFQG) in place of the second separase site (SVEQGRR), and Scc3-FLAG were grown in YP medium containing 2% raffinose as the carbon source to an optical density of 1.0 at 30 °C. 2% galactose was then added to the culture to induce protein expression, and cells were further grown for 2 hours. Cells were collected by centrifugation, washed with deionized water, and suspended in cohesin buffer containing 300 mM NaCl, 0.5 mM TCEP, 2 mM MgCl_2_, 0.5 mM Pefabloc, as well as cOmplete-EDTA protease inhibitor cocktail. The cell suspension was frozen in liquid nitrogen, then cells were broken in a cryogenic freezer mill. The cell powder was thawed on ice, and further cohesin buffer containing 300 mM NaCl, 0.5 mM TCEP, 0.5 mM MgCl_2_ and protease inhibitors was added. The lysate was clarified by centrifugation at 20,000 x g for 1 hour. CaCl_2_, RNase A, and benzonase were added to final concentrations of 2 mM, 0.3 μg/ml, and 1.25 U/ml, respectively. The lysate was transferred to pre-equilibrated CBP affinity resin and incubated for 2 hours. The resin was washed with cohesin buffer containing 300 mM NaCl, 0.5 mM TCEP, 2 mM CaCl_2_ and 2 mM MgCl_2_, then incubated in cohesin buffer containing 300 mM NaCl, 0.5 mM TCEP, 2 mM CaCl_2_, 10 mM MgCl_2_ and 1 mM ATP for 15 minutes. The resin was washed again with cohesin buffer containing 300 mM NaCl, 0.5 mM TCEP, 2 mM CaCl_2_, and 2 mM MgCl_2_, then incubated in cohesin buffer containing 300mM NaCl, 0.5 mM TCEP, 2 mM EDTA, and 2 mM EGTA to elute the proteins. The eluate was collected and further purified using HiTrap Heparin and Superose 6 Increase 10/300 GL chromatography, as described above for 6C Cohesin.

#### Chlorella virus Top II purification

During the course of our experiments, we noticed that budding yeast Top II interferes with DNA replication in reactions carried out at lower salt concentrations. As an alternative to yeast Top II, we purified *Chlorella* virus Top II^53^ as described below. The *Chlorella* virus enzyme proficiently supported DNA replication at low salt concentrations and was used in these experiments.

Budding yeast cells overexpressing *Chlorella* virus Top II were grown in YP medium containing 2% raffinose as the carbon source to an optical density of 1.0 at 30 °C. 2% galactose was then added to the culture to induce protein expression, and cells were further grown for 2 hours. Cells were collected by centrifugation, washed with deionized water, and suspended in CV Top II buffer (25 mM Tris-HCl pH 7.2, 10% glycerol, 400 mM NaCl, 0.01% NP-40, 1 mM DTT) containing 0.5 mM Pefabloc, as well as cOmplete-EDTA protease inhibitor cocktail. The cell suspension was frozen in liquid nitrogen, then cells were broken in a cryogenic freezer mill. The cell powder was thawed on ice, and further CV Top II buffer containing protease inhibitors was added. The lysate was clarified by centrifugation at 20,000 x g for 1 hour. RNase A was added to a final concentration of 0.3 μg/ml to the clarified lysate. The lysate was transferred to pre-equilibrated anti-FLAG M2 affinity gel and incubated for 2 hours. The resin was washed with CV Top II buffer, then incubated in the same buffer containing 10 mM MgCl_2_ and 1 mM ATP for 15 minutes. The resin was washed again with CV Top II buffer, then incubated in the same buffer containing 0.5 mg/ml FLAG peptide. The eluate was loaded onto a Superdex 200 Increase 10/300 GL gel filtration column that was equilibrated and developed with CV Top II gel filtration buffer (25 mM Tris-HCl pH 7.2, 150 mM NaCl, 10% Glycerol, 0.01% NP-40, 1 mM DTT). The peak fractions were combined.

#### Chl1 purification

Codon optimized budding yeast Chl1 was expressed and purified as previously described.^54^ We noticed that increased Chl1 yield due to codon optimization led to less favorable protein behavior during purification. Alternatively, therefore, we expressed Chl1 encoded by its native sequence as detailed below. Once purified, both Chl1 preparations showed similar behavior and activity, and they were used interchangeably in this study.

Budding yeast cells overexpressing Chl1 from its native sequences were grown in YP medium containing 2% raffinose as the carbon source to an optical density of 1.0 at 30 °C. 2% galactose was added to the culture to induce protein expression, and cells were further grown for 3 hours. Cells were collected by centrifugation, washed with deionized water, and suspended in Chl1 buffer (20 mM HEPES-KOH pH 7.5, 20% glycerol, 300 mM NaCl, 0.01% Tween-20, 0.5 mM TCEP) containing 0.5 mM Pefabloc, as well as SIGMAFAST Protease Inhibitor Cocktail EDTA-free. The cell suspension was frozen in liquid nitrogen, then cells were broken in a cryogenic freezer mill. The cell powder was thawed on ice, and further Chl1 buffer containing protease inhibitors was added. The lysate was clarified by centrifugation at 20,000 x g for 1 hour. The lysate was transferred to pre-equilibrated anti-FLAG M2 affinity gel and incubated for 2 hours. The resin was washed with Chl1 buffer, then incubated in the same buffer containing 10 mM MgCl_2_ and 1 mM ATP for 15 minutes. The resin was washed again with Chl1 buffer, then incubated in the same buffer containing 0.5 mg/ml FLAG peptide. The eluate was loaded onto a Superdex 200 Increase 10/300 GL gel filtration column that was equilibrated and developed with Chl1 gel filtration buffer (20 mM HEPES-KOH pH 7.5, 200 mM NaCl, 10% Glycerol, 0.5 mM TCEP). The peak fractions were pooled.

To assess Chl1 helicase activity, we prepared a forked DNA substrate by mixing equal amount of rhodamine-labeled oligonucleotide MM527 (5’-[RhoX]GTGGTGAGGAGAGGTCAGTGCTGCGGCTGGTTTTTTTTTTTTTTTTTTTTTTTTTTTTTTTTTTTTTTTT-3’) and unlabeled oligonucleotide MM528 (5’-TTTTTTTTTTTTTTTTTTTTTTTTTTTTTTTTTTTTTTTTCCAGCCGCAGCACTGACCTCTCCT CACCAC-3’) heating to 90 °C, and gradual cooling to room temperature. The resultant substrate (20 nM) was incubated with Chl1 in helicase buffer (25 mM HEPES/KOH pH 7.5, 0.1 mg/ml BSA, 2 mM magnesium acetate, 5 mM ATP), in a reaction volume of 20 μl at 30 °C for 15 minutes, 8 μl of stop buffer (10 mM HEPES/KOH pH 7.5, 1% SDS, 50 mM EDTA, 1 mg/ml proteinase K) containing 100 nM of an oligonucleotide MM529 (5’-GTGGTGAGGAGAGGTCAGTGCTGCGGCTGG-3’) was added to quench the reaction, and further incubated at 30 °C for 15 minutes. Reactions were then resolved by 10% PAGE in TBE, and rhodamine on MM527 was visualized using a ChemiDoc MP Imaging system (BioRad).

To measure DNA-dependent ATP hydrolysis activity of Chl1, 100 nM Chl1 was combined with 10 nM circular, 3.2 kb dsDNA or ssDNA in buffer containing 25 mM HEPES/KOH pH 7.5, 250 mM potassium glutamate, 10 mM magnesium acetate, 0.01% NP40, 1 mM DTT. The reaction was initiated by the addition of 1 mM ATP, spiked with [γ-^32^P]-ATP, and incubated at 30 °C. Reaction aliquots were retrieved at 10, 20, 30 and 40 minutes and terminated by adding 375 mM EDTA. 1 μl aliquots of the reactions were spotted onto polyethylenimine cellulose F sheets and separated by thin-layer chromatography using 0.75 M KH_2_PO_4_ (pH 3.4) as the mobile phase. The separated spots representing ATP and released inorganic phosphate were quantified using a Typhoon FLA 9500 Imager and Fiji.

#### DNA substrates

pBluescript-based DNA replication substrates, harboring the *ARS1* replication origin and a size of 3.2 kb or 5.8 kb^33^ were prepared from *E. coli* using the QIAGEN Plasmid Mega Kit. While the resultant preparation majorly consists of monomeric, covalently closed circular DNA, a minority of nicked or catenated species are observed, which were removed as follows. To eliminate nicked DNA, the preparation was treated with T5 exonuclease, and the reaction quenched by addition of SDS to a final concentration of 1%. The DNA was then loaded onto a 10 - 25% sucrose gradient, in gradient buffer (50 mM Tris-HCl pH 8, 2 mM EDTA, 50 mM NaCl, 0.1% Triton X-100) prepared in 5 ml Open-Top Thinwall Ultra-Clear Tubes. The tubes were centrifuged at 73,000 x g for 18 hours (for the 3.2 kb plasmid) or at 58,000 x g for 16 hours (for the 5.8 kb plasmid). 0.3 ml fractions were then harvested manually. Monomeric DNA peak fractions were identified by agarose gel electrophoresis, and the DNA purified and recovered using phenol-chloroform extraction and ethanol precipitation.

#### Cohesion establishment using pre-loaded cohesin

All incubations were conducted at 30 °C. Cohesin loading was performed in CL buffer (25 mM HEPES-KOH pH 7.5, 10% Glycerol, 0.5 mM MgCl_2_, 0.003% NP-40, 2 mM TCEP, 20 mM potassium glutamate, 5 mM ATP). Circular DNA, cohesin and the Scc2-Scc4 cohesin loader complex were added to final concentrations of 3.3 nM, 120 nM and 60 nM, respectively. The reaction proceeded for 30 minutes and then 1.5 times diluted in MCM loading buffer (25 mM HEPES-KOH pH 7.5, 30 mM magnesium acetate, 2 mM TCEP, 0.02% NP-40, 250 mM potassium glutamate, 10 mM ATP). ORC, Cdt1-Mcm2-7 and Cdc6 were added at a final concentration of 10 nM, 30 nM, 50 nM, respectively. After 15 minutes incubation, DDK and S-CDK were added at a final concentration of 40 nM and 20 nM, respectively. After further 15 minutes, the reaction was twofold diluted in replication buffer (25 mM HEPES-KOH pH 7.5, 300 mM potassium glutamate, 10 mM magnesium acetate, 0.02% NP-40, 2.4 mM ATP, 400 μM each of TTP, GTP and CTP, 80μM each of dATP, dTTP, dGTP and dCTP, 2 mM TCEP, 1 mM acetyl-CoA, 66 nM [α-^32^P]dCTP). If not indicated otherwise, 100 nM RPA, 30 nM Dpb11, 40 nM Cdc45, 5 nM GINS, 10 nM S-CDK, 10 nM Top I, 20 nM Ctf4, 40 nM Chl1, 20 nM Polε, 5 nM Mcm10, 20 nM Tof1-Csm3, 20 nM Mrc1, 40 nM RFC, 80 nM PCNA, 0.4 nM Pol δ, 40 nM Fen1, 60 nM Cdc9, 50 nM Pds5, 40 nM\ Eco1, 10 nM Dna2, 25 nM Pol α, 65 nM Sld2, 25 nM Sld3-Sld7, 10 nM Ctf18-RFC, 5 nM Pif1, and either 2.5 nM budding yeast Top II or 2 nM *Chlorella* virus Top II were added, and replication reactions were incubated for 40 minutes.

#### Cohesin immunoprecipitation (without crosslinking)

8 μl of a replication reaction was taken as the DNA input sample. 12 μl deproteination buffer (10 mM Tris-HCl pH 7.5, 1 mM EDTA, 50 mM NaCl, 0.75% SDS) containing 1.7 mg/ml proteinase K were added, and incubated at 37 °C for 20 minutes. 80 μl replication reaction were used to immunoprecipitate cohesin. 250 μl of IP buffer 1 (25 mM HEPES-KOH pH 7.5, 500 mM NaCl, 0.1% NP-40, 5 mM EDTA) were added together with α-Pk antibody-coated protein A-conjugated magnetic beads. The mix was incubated on ice with gentle agitation for 40 minutes, and then washed extensively with IP buffer 1. The magnetic beads were then suspended in 20 μl of deproteination buffer containing 1 mg/ml proteinase K, and incubated at 37 °C for 20 minutes.

The input and immunoprecipitated DNA fractions were phenol-chloroform extracted, then loaded onto MicroSpin G-50 Columns, equilibrated with Nb.BssSI buffer (50 mM Tris-HCl pH 8, 100 mM NaCl, 10 mM MgCl_2_, 0.02% NP-40). To nick DNA, if applicable, Nb. BssSI was added to and incubated for 20 minutes at 37 °C. The DNA was resolved by 0.8% agarose gel electrophoresis in TAE buffer and visualized using SYBR Gold Nucleic Acid Gel Stain and a ChemiDoc MP Imaging system (BioRad). To visualize replication products that incorporated [α-^32^P]dCTP, the same agarose gel was dried, exposed to an Imaging Plate (Fujifilm), and scanned using a Typhoon FLA 9500 biomolecular imager (Cytiva).

#### 6C Cohesin crosslinking, denaturation and immunoprecipitation

DNA replication was conducted essentially as described above, but [α-^32^P]dCTP was used at a final concentration of 250 nM, and replication reaction proceeded for 50 minutes. Cdc9 was not included in the experiments shown in Figures 2B, 3D, and 4B (to yield nicked replication products). After replication, 0.5 mM bis-maleimidoethane BMOE was added to 6C (or 5C) cohesin containing reactions. Crosslinking proceeded for 20 minutes at room temperature. The reaction was quenched by addition of 2 mM DTT. For protein denaturation, SDS was added to a final concentration of 1% before incubation at 65 °C for 20 minutes. The SDS-containing samples were diluted by tenfold using IP dilution buffer (25 mM Tris pH8, 150 mM NaCl, 1% NP-40, 20 mM EDTA), and BSA was added to a final concentration of 0.5 mg/ml. α-Pk antibody-coated protein A-conjugated magnetic beads were added, and the samples kept on ice with gentle agitation for 60 minutes. The magnetic beads were washed using wash buffer 1 (50mM Tris-HCl pH8, 500 mM NaCl, 10 mM EDTA, 0.1% NP-40). As applicable, to nick recovered DNA, the cohesin-DNA complexes on magnetic beads were further treated with Nb.BssSI buffer containing 200 U/ml Nb.BssSI for 15 minutes at 30 °C. To analyze the DNA methylation status, the cohesin-DNA complexes on magnetic beads were further treated in Dpn I buffer (50 mM Tris-HCl pH 8, 300 mM NaCl, 10 mM MgCl_2_, 0.05% NP-40) containing 200 U/ml Dpn I for 15 minutes at 30 °C. Cohesin-bound replication products were then eluted from the antibody beads in buffer containing 10 mM Tris-HCl pH 8, 1 mM EDTA, 100 mM NaCl, and 1% SDS at 65 °C.

#### Two-dimensional (2D) agarose gel electrophoresis

6C cohesin-replicated DNA complexes were firstly resolved at 1.4V/cm on a 0.7% agarose TAE gel containing 0.2% SDS for 15 hours (first dimension). Lanes from the gel were cut and placed at the top of a second 0.7% agarose TAE gel containing 0.2% SDS, leaving an approximately 1.5 cm wide gap between the gel lane and the new gel. The gap was filled with 60 °C 0.7% agarose in TAE, containing 0.2% SDS and 0.2 mg/ml proteinase K. Once the molten agarose solution solidified, the 6C cohesin-replicated DNA complexes were again resolved at 1.4V/cm for 15 hours, where 6C cohesin was digested by proteinase K (second dimension). 0.2% SDS was included in the TAE running buffer for both first and second dimension. The agarose gel was dried, exposed to an imaging plate and scanned as above.

#### Replication-coupled cohesin acetylation

30 nM cohesin was incubated with 3.3 nM template DNA and 60 nM Scc2-Scc4 in CL buffer for 60 minutes at 30 °C. The cohesin loading reaction was terminated by adding equal volume of IP buffer 2 (35 mM Tris-HCl pH 7.5, 180 mM NaCl, 20 mM EDTA, 10% glycerol, 0.7% Triton X-100). anti-myc antibody-coated protein G-conjugated magnetic beads were added to the reaction, then the samples were rocked for 60 minutes at 4 °C. The beads were washed in wash buffer 3 (35 mM Tris-HCl pH 7.5, 500 mM NaCl, 10 mM EDTA, 5% glycerol, 0.35% Triton X-100), and then in wash buffer 4 (35 mM Tris-HCl pH 7.5, 100 mM NaCl, 0.1% Triton X-100). The beads were suspended in the same buffer containing 0.125 mg/ml myc peptide and incubated at 25 °C for 40 minutes. The eluate was loaded onto MicroSpin G-50 columns, equilibrated with MCM loading buffer (25 mM HEPES-KOH pH 7.5, 10 mM magnesium acetate, 0.01% NP-40, 100 mM potassium glutamate) containing 1 mM DTT. ATP was then added to a final concentration of 5 mM, and MCM loading and DNA replication were performed as above. Following the replication incubation, SDS sample buffer was added to the reaction and boiled. Proteins were separated using SDS-PAGE and transferred to a nitrocellulose membrane for immunoblotting.

#### Spontaneous cohesin loading without the cohesin loader

50 nM cohesin was incubated with 3.3 nM circular DNA in CL buffer for 60 minutes at 30 °C. The cohesin loading reaction was terminated by adding equal volume of IP buffer 2 (35 mM Tris-HCl pH 7.5, 180 mM NaCl, 20 mM EDTA, 10% glycerol, 0.05% NP-40). anti-FLAG M2 antibody-coated protein G-conjugated magnetic beads were added to the reaction, then the samples were rocked for 60 minutes at 4 °C. The magnetic beads were washed in wash buffer 5 (25 mM HEPES-KOH pH 7.5, 500 mM NaCl, 0.02% NP-40, 5 mM EDTA, 1 mM TCEP), and then in MCM loading buffer (25 mM HEPES-KOH pH 7.5, 10 mM magnesium acetate, 0.01% NP-40, 100 mM potassium glutamate) containing 2 mM TCEP. The beads were suspended in the same buffer containing 0.1 mg/ ml FLAG peptide, and incubated at 30 °C for 5 minutes. The beads were removed using a magnet, ATP was added to a final concentration of 5 mM, and MCM loading, DNA replication, cohesin crosslinking, protein denaturation, and cohesin immunoprecipitation were performed as above.

#### Replication-coupled cohesin loading

4 nM 3.5 kb template DNA, 10 nM ORC, 50 nM Cdc6 and 30 nM Cdt1-Mcm2-7 were mixed in MCM loading buffer containing 0.5 mM TCEP and 5 mM ATP and incubated at 30 °C for 15 minutes. DDK and S-CDK were then added to a final concentration of 50 nM and 20 nM, respectively. After 15 minutes incubation, the reaction was twofold diluted into low-salt replication buffer (25 mM HEPES-KOH pH 7.5, 100 mM potassium glutamate, 10 mM magnesium acetate, 0.02% NP-40, 2.4 mM ATP, 400μM each of TTP, GTP and CTP, 80 μM each of dATP, dTTP, dGTP and dCTP, 2 mM TCEP, 1 mM acetyl-CoA, 66 nM [α-^32^P]dCTP). 100 nM RPA, 30 nM Dpb11, 40 nM Cdc45, 5 nM GINS, 10 nM S-CDK, 20 nM Top I, 20 nM Ctf4, 40 nM Chl1, 20 nM Polε, 5 nM Mcm10, 20 nM Tof1-Csm3, 20 nM Mrc1, 40 nM RFC, 80 nM PCNA, 25 nM Pol α, 65 nM Sld2, 25 nM Sld3-Sld7 were also added, together with 50 nM Cohesin and 30 nM Scc2-Scc4. The replication reaction proceeded for 20 minutes. (5 nM Pif1 was included and replication reactions extended to 40 minutes to facilitate quantitation of completely replicated DNA circles in the experiments shown in Figure S6). After replication, a 7 μl aliquot was taken as the DNA input sample. 1% SDS and 2 mg/ml proteinase K final concentrations were added and the aliquots incubated at 37 °C for 20 minutes. To analyze cohesin-bound DNA, 170 μl of IP buffer 1 were added to 28 μl of the remaining replication reactions, together with α-Pk antibody-coated protein A-conjugated magnetic beads. Immunoprecipitation was conducted for 40 minutes. The magnetic beads were washed with the IP buffer 1, then suspended in MCM loading buffer containing 1% SDS and 2 mg/ml proteinase K at 37 °C for 20 minutes.

The input and cohesin-bound DNA fractions were phenol-chloroform extracted and analyzed by agarose gel electrophoresis in TAE buffer. SYBR-Gold staining and autoradiography were performed as above.

#### Cohesion establishment during replication-coupled cohesin loading

To analyze the establishment of sister chromatid cohesion by replication-coupled cohesin loading, 6C-cohesin was used and 0.4 nM Pol δ, 40 nM Fen1, 60 nM Cdc9, 50 nM Pds5, 40 nM Eco1, 10 nM Dna2, 10 nM Pif1, 10 nM Ctf18-RFC, and 1.25 nM *Chlorella* virus Top II were supplemented to the above replication-coupled cohesin loading reactions. The reactions were incubated for 50 minutes. BMOE crosslinking, protein denaturation, cohesin immunoprecipitation, Nb.BssSI nicking enzyme treatment, Dpn I treatment, and 2D agarose gel electrophoresis were then performed as described above.

### Quantification and Statistical Analysis

Conclusions in this study that are based on qualitative comparisons were confirmed by experimental repeats. In these cases, representative experiments are shown and details on the number of repeat experiments can be found in the figure legends. In cases where a quantitative comparison was required, three independent repeat experiments were performed. In these experiments, cohesin-associated total DNA and replication products were detected and quantified by SYBR Gold staining and autoradiography, respectively, using a Typhoon FLA 9500 biomolecular imager (Cytiva). Individual results from all three repeats are shown, together with the means and standard deviations.

## Supplementary Material

Supplemental information can be found online at https://doi.org/10.1016/j.molcel.2025.08.026.

Supplementary Materials

## Highlights

- Biochemical reconstitution of sister chromatid cohesion establishment
- Cohesin retains DNA embrace throughout DNA replication
- New cohesin recruitment also leads to sister chromatid cohesion
- More often than not, cohesin entraps only one of the two DNA products

## In brief

Genome inheritance during cell divisions relies on replicated sister DNAs being connected by cohesin rings. Minamino et al. reconstitute sister chromatid cohesion establishment during DNA replication using purified budding yeast proteins. The experiments yield molecular insight into more than one way in which sister chromatid cohesion can be built.
