# Supplementary material for: Biochemical reconstitution of sister chromatid cohesion establishment during DNA replication
Source: Mol Cell. Author manuscript; Available in PMC 2026 Aug 26. (PMC7619402; doi:10.1016/j.molcel.2025.08.026)
Supplement: Supplementary file 1 [file EMS217825-supplement-Supplementary_Materials.pdf]

Molecular Cell, Volume *85*

Supplemental information

Biochemical reconstitution of sister chromatid cohesion establishment during DNA replication

Masashi Minamino, John F.X. Diffley, and Frank Uhlmann

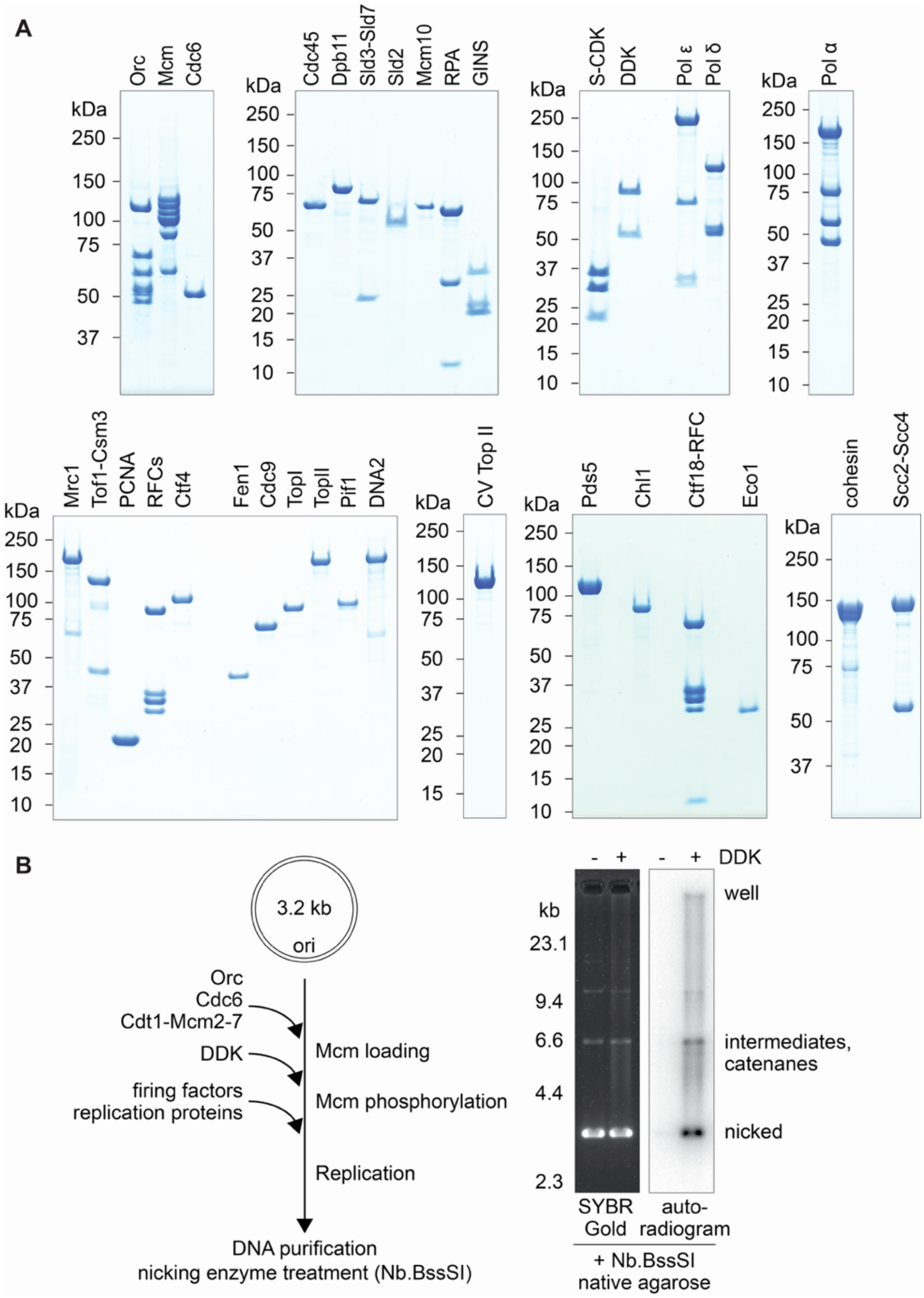

Figure S1. Characterization of the DNA replication reaction, Related to Figure 1
(A) Purified replication and sister chromatid cohesion factors used in this study were analyzed by SDS-PAGE followed by Coomassie Blue staining.
(B) Schematic of a complete plasmid DNA replication reaction, and example gel electrophoretic analysis of the total DNA visualized with the DNA stain SYBR Gold and of the replication products visualized by [α-^{32}P]dCTP autoradiography. While our input template DNA is supercoiled, topoisomerases present in the replication reaction led to its partial relaxation. To facilitate the comparison between input and replicated DNA, irrespective of their supercoiling state, we treated all samples with a nicking enzyme before agarose gel electrophoresis that converts them into relaxed circles. The experiment demonstrates the dependence of DNA replication on the DDK origin activation kinase.

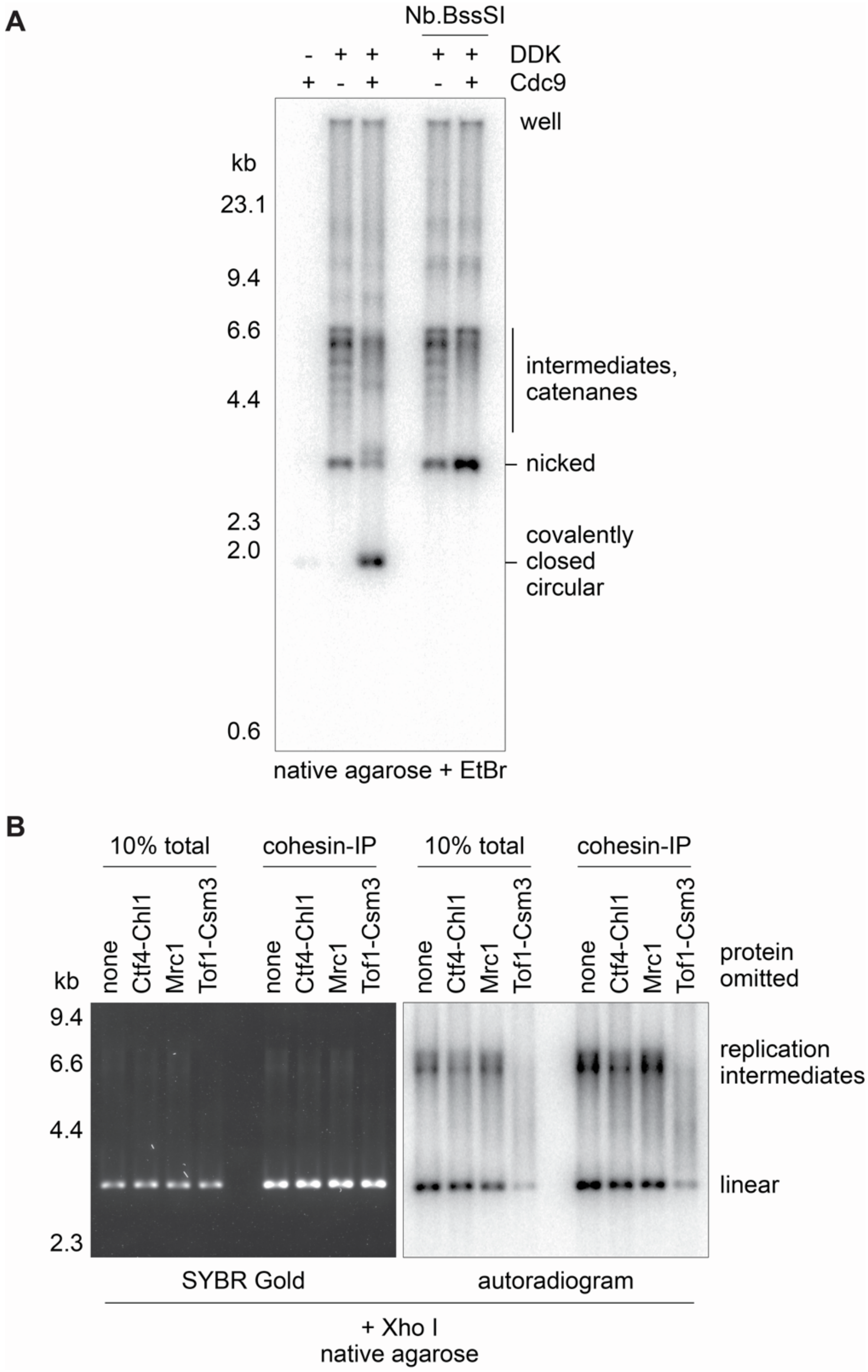

Figure S2. Characterization of the fully replicated species, and an experiment to analyze the contribution of cohesion establishment factors to cohesin retention during DNA replication, Related to Figure 1

(A) Evidence that the fast-migrating population of replication products, detected during agarose gel electrophoresis in the presence of ethidium bromide (EtBr), consists of fully replicated, covalently closed circular DNAs. The species does not form in the absence of the DNA ligase Cdc9, required to seal nicks that remain between processed Okazaki fragments (left). Furthermore, nicking of the product by Nb.BssSI treatment following DNA purification prevents supercoiling by EtBr intercalation.

(B) The indicated cohesion establishment factors were omitted from DNA replication reactions. Total DNA following DNA replication, as well as cohesin-bound fractions were analyzed. Replication products were visualized by [α-^{32}P]dCTP autoradiography. To facilitate comparisons, DNAs were cleaved with the restriction enzyme XhoI before gel electrophoresis, resulting in a single band representing fully replicated products. Note that, as expected [S1], the efficiency of DNA replication was markedly reduced in the absence of Tof1-Csm3.

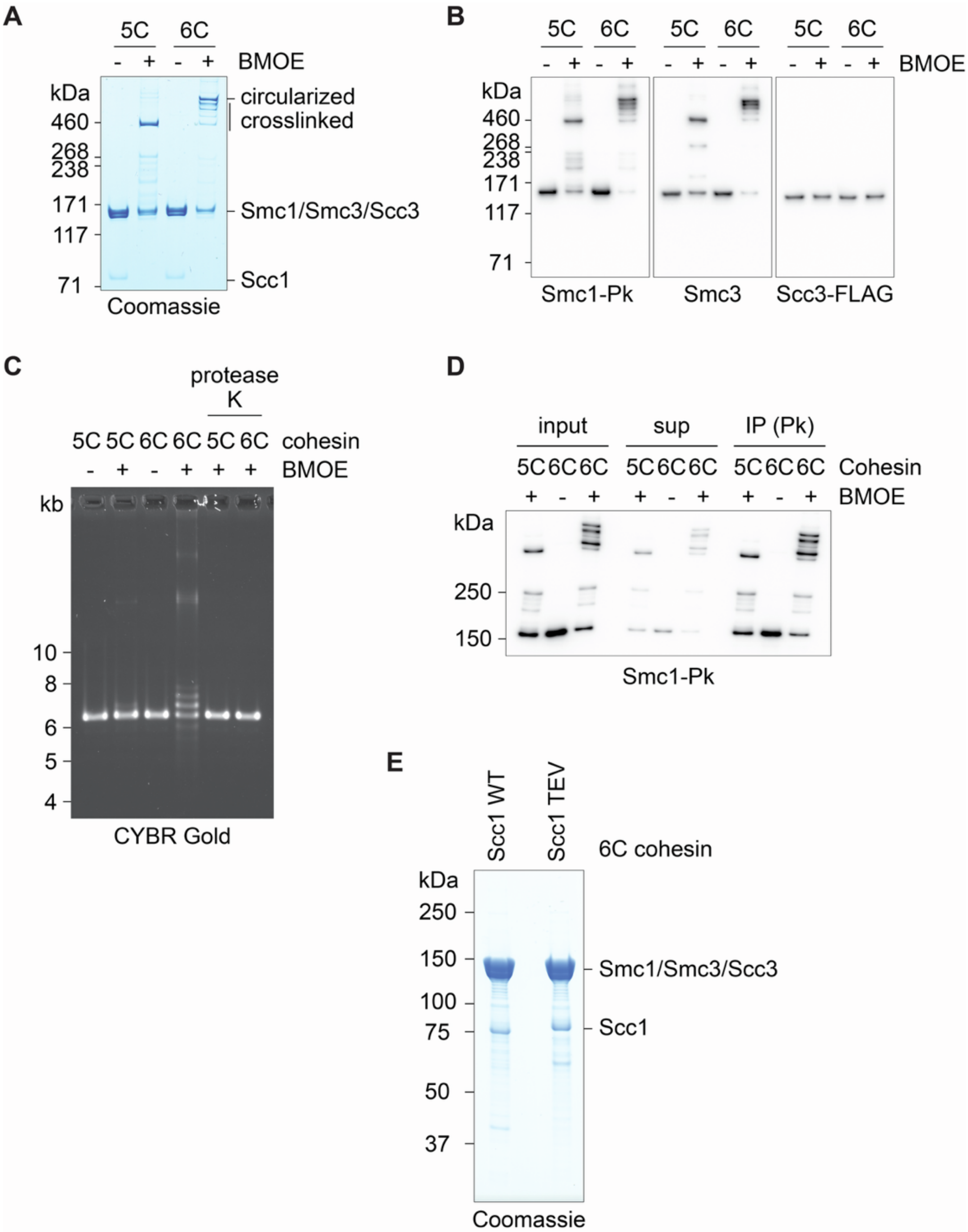

Figure S3. Characterization of 6C cohesin crosslinking, Related to Figure 2
(A) 5C and 6C cohesin were incubated in the absence or presence of bis-maleimidoethane (BMOE) and analyzed by SDS-PAGE followed by Coomassie staining.
(B) Identification of the crosslinked species. As (A), but immunoblotting was used to detect the indicated subunits using their respective antibodies.
(C) Topological DNA entrapment by 6C cohesin. 5C and 6C cohesin were loaded onto the circular template DNA in presence of the cohesin loader, followed by BMOE crosslinking as indicated, and SDS denaturation. Where indicated proteinase K was included before the DNA was resolved by agarose gel electrophoresis.
(D) 5C and 6C cohesin recovery by immunoprecipitation in the experiment shown in Figure 2A. Equal portions of the input, supernatant (sup) and immunoprecipitate (IP) fractions were analyzed by immunoblotting.

(E) Purified 6C cohesin containing wild type Scc1 (WT), or Scc1 in which one of the separase recognition sites was exchanged for the TEV protease recognition sequence (TEV) [S2] were analyzed by SDS-PAGE followed by Coomassie staining.

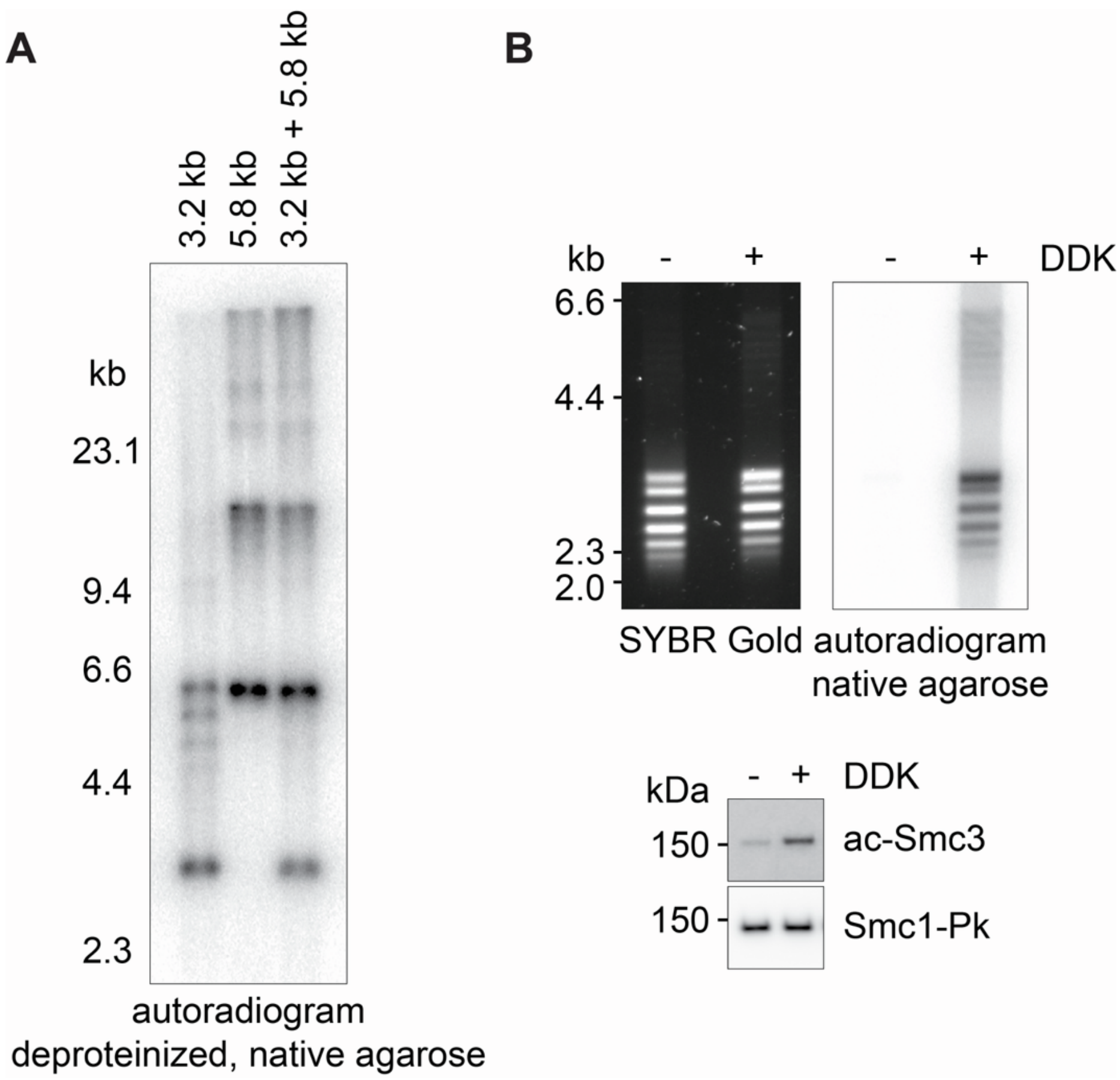

Figure S4. Further characterization of *in vitro* cohesion establishment, Related to Figure 3

(A) Replication reactions were performed with either or both of two circular, *ARS1*-containing, DNA templates 3.2 kb and 5.8 kb in size. Replication products were analyzed following deproteinization by agarose gel electrophoresis and autoradiography. When combined, both templates were replicated with approximately equal efficiencies.

(B) Replication-coupled cohesin acetylation. Cohesin was loaded onto template DNA, then immunopurified and peptide-eluted to enrich cohesin-bound templates. Replication incubations were performed with or without DDK. Total DNA and replication products were visualized, and an aliquot of the reaction was processed for immunoblotting using an acetylated Smc3-specific antibody. The Pk epitope-tagged cohesin subunit Smc1 served as a loading control.

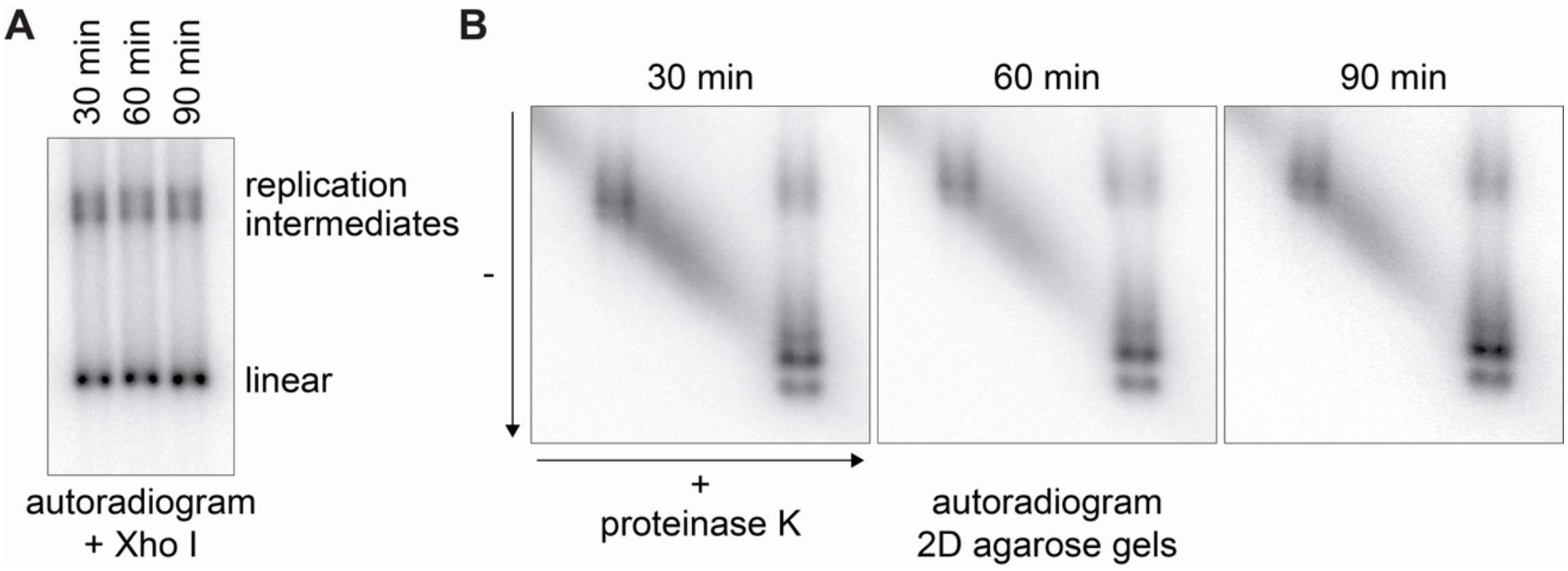

Figure S5. Timecourse analysis to probe the stability of *in vitro* established sister chromatid cohesion, Related to Figure 4
(A) Replication reactions using 6C cohesin-bound templates were incubated for the indicated times. The replication products were analyzed following deproteinization and restriction enzyme cleavage using XhoI. This analysis reveals that replication has largely reached completion after 30 minutes.
(B) Aliquots from the replication reactions in (A) were BMOE crosslinked, denatured, and cohesin-bound DNAs were enriched by cohesin immunoprecipitation. 2D gel electrophoresis reveals that the fraction of cohesin that promotes sister chromatid cohesion, or associates with only one of two replication products, remains approximately constant over time.

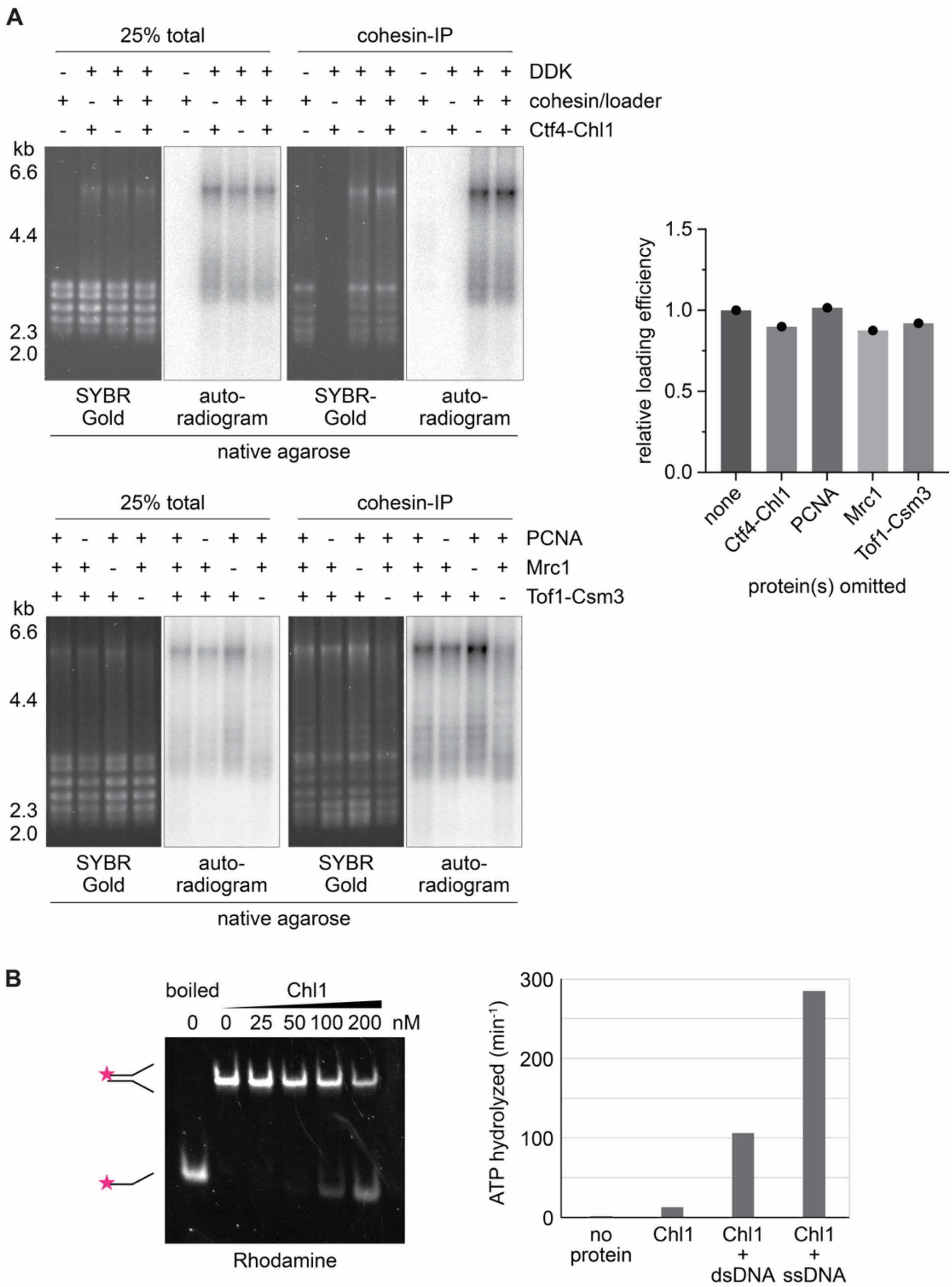

Figure S6. Characterization of replication-coupled cohesin loading, Related to Figure 5

(A) Replication reactions were performed in the presence of cohesin with or without the indicated factors. Total and cohesin-bound DNAs were afterwards analyzed by agarose gel electrophoresis. SYBR Gold stained all DNA, while autoradiography was used to detect replication products. The replication-coupled cohesin loading efficiency was quantified as the fraction of replication products retrieved by cohesin immunoprecipitation and is shown relative to a reaction in which no protein was omitted.

(B) Characterization of the Chl1 helicase preparation used in our study. Both the DNA unwinding and DNA-stimulated ATP hydrolysis activities (indicated as ATPs hydrolyzed per Chl1 molecule) of the enzyme are comparable to that observed by Murayama et al. [S3]. See the Methods for reaction condition details.

Supplemental References

S1. Yeeles, J.T.P., Janska, A., Early, A., and Diffley, J.F.X. (2017). How the eukaryotic replisome achieves rapid and efficient DNA replication. Mol. Cell *65*, 105-116.

S2. Uhlmann, F., Wernic, D., Poupart, M.-A., Koonin, E.V., and Nasmyth, K. (2000). Cleavage of cohesin by the CD clan protease separin triggers anaphase in yeast. Cell *103*, 375-386.

S3. Murayama, Y., Endo, S., Kurokawa, Y., Kurita, A., Iwasaki, S., and Araki, H. (2024). Coordination of cohesin and DNA replication observed with purified proteins. Nature *626*, 653-660.
